# Human sensory-evoked responses differ coincident with either "fusion-memory" or "flash-memory", as shown by stimulus repetition-rate effects

**DOI:** 10.1186/1471-2202-7-18

**Published:** 2006-02-23

**Authors:** Don L Jewett, Toryalai Hart, Linda J Larson-Prior, Bill Baird, Marram Olson, Michael Trumpis, Katherine Makayed, Payam Bavafa

**Affiliations:** 1 Abratech Corporation, Sausalito, CA, USA.; 2 Radiology Dept., Washington University, St. Louis, MO, USA.; 3 Neurotechnology Research & Consulting, Oakland, CA, USA.; 4 Emeritus Professor, University of California, San Francisco, USA.

## Abstract

**Background:**

A new method has been used to obtain human sensory evoked-responses whose time-domain waveforms have been undetectable by previous methods. These newly discovered evoked-responses have durations that exceed the time between the stimuli in a continuous stream, thus causing an overlap which, up to now, has prevented their detection. We have named them "A-waves", and added a prefix to show the sensory system from which the responses were obtained (visA-waves, audA-waves, somA-waves).

**Results:**

When A-waves were studied as a function of stimulus repetition-rate, it was found that there were systematic differences in waveshape at repetition-rates above and below the psychophysical region in which the sensation of individual stimuli fuse into a continuity. The fusion phenomena is sometimes measured by a "Critical Fusion Frequency", but for this research we can only identify a frequency-region [which we call the STZ (Sensation-Transition Zone)]. Thus, the A-waves above the STZ differed from those below the STZ, as did the sensations.

Study of the psychophysical differences in auditory and visual stimuli, as shown in this paper, suggest that different stimulus features are detected, and remembered, at stimulation rates above and below STZ.

**Conclusion:**

The results motivate us to speculate that:

1) Stimulus repetition-rates above the STZ generate waveforms which underlie "fusion-memory" whereas rates below the STZ show neuronal processing in which "flash-memory" occurs.

2) These two memories differ in both duration and mechanism, though they may occur in the same cell groups.

3) The differences in neuronal processing may be related to "figure" and "ground" differentiation.

We conclude that A-waves provide a novel measure of neural processes that can be detected on the human scalp, and speculate that they may extend clinical applications of evoked response recordings. If A-waves also occur in animals, it is likely that A-waves will provide new methods for comparison of activity of neuronal populations and single cells.

## Background

### The sensation transition-zone for fusion

A well established psychophysical effect is the change in sensation when repetition-rate is increased to the point where previously-sensed "individual stimuli" become "fused". In vision, flashed "stop-action" becomes a "movie" at higher flash-rates, while in audition, the sensation of "discrete sounds" comes to contain a "musical tone" when the same transient sound is repeated above about 20 S/s (Stimuli/sec). The early history of research on fusion has been summarized in these words by Kompass [1] :

"The first, to my knowledge, empirical contribution to this line of research was given by Lalanne (in 1876: [2]) who pointed out that the frequency of stimulus fusion in the tactile, auditory, and visual modality equals 18 Hz. Lalanne conjectured a common, yet unknown, mechanism behind this.

"Measuring tactile stimulus fusion, Brecher (in 1932: [3]) found that the critical frequency did not depend on intensity of stimulation or the cutaneous receptor density: Stimulation of the tips of tongue and fingers gave approximately the same critical frequency value as stimulation of the back or the feet. Variability between participants was very small: Individual averages of 14 participants yielded anoverall mean period of 55.3 ms (18.1 Hz) and a standard deviation of 1.2 ms between participants. This seemed surprising because it was known that other well-determinable psychological constants such as Weber fractions differ much more among participants."

A commonly-used term in psychophysiology is CFF (Critical Fusion Frequency) for the sensory transition. CFF rate has been studied as an indicator of arousal and attention and has had clinical use as a diagnostic tool for multiple sclerosis, migraine, Altzheimer's, Parkinson's and other diseases [4-11]. We will describe differences in evoked-responses as a function of stimulus repetition-rate, in which *qualitatively different *evoked-responses occur at repetition-rates below and above what we will call the STZ (Sensation-Transition Zone). We use the term STZ rather than CFF because referring to a rate-boundary between two phenomena in the singular implies that a single rate can be identified, and is unchanging. But a given endpoint may be affected by hysteresis, as was noted by von Bekesy [12,13], who also found a range of auditory endpoints if intensity was held constant and frequency varied. Furthermore, in vision the CFF varies as a function of position in the visual field. For our purposes now, it is better to define the STZ as a psychophysical region where the stimulus-repetition rate may not be precisely known, may not be constant, and may depend on other stimulus parameters. We have studied stimulus repetition-rates that are on either side of the STZ. Thus, we can only describe a range of stimulus repetition-rates in which the transition occurs, not "***the ****boundary*".

A note on terminology: Since stimuli can be non-sinusoidal transients, for stimulus repetition-rate we use the units of Stimuli per second (i.e., 10 S/s). If we are referring to sinusoidal waveforms (as in the Frequency Domain), we use Hz as the units.

### Technical limitations in experimentation with continuously-repeating transient stimuli

Evoked-response recordings that produce temporal waveforms have been limited to repetition-rates that provide an SI (Stimulus Interval, ***start****-to-start*) which is *longer than the observed evoked-response waveform *(using appropriate filtering). The consequence is that high stimulus repetition-rates have not been studied, except by means of SS (Steady-State) responses, which have important limitations (described next and in the Discussion).

SS responses are obtained using a uniform repetition-rate, which makes *recovery of any *time-domain *transient brain-response waveform *to each stimulus ***mathematically impossible***. (For proof of this statement, see our paper on QSD [14].) SS evoked responses measure only the magnitude and phase of the Fourier coefficients at the stimulus repetition-rate and its integer multiples. The limitations caused by measuring only the magnitude of the Fourier coefficient (often only at the stimulus repetition-rate) may be the reason that *SS evoked potentials *in the auditory and visual systems [15,16] show *no change in electrical potentials that correspond to the CFF*. In vision, van der Tweel et al. [17] looked specifically for a connection between sinusoidal SSVEPs (Steady-State Visual Evoked Potentials) and the CFF boundary measured as a function of both stimulation rate and modulation depth. They concluded that "the lack of correspondence between the results of the psychophysical studies and those obtained in electrophysiology is striking". Other studies also report a *lack of correlation *between evoked-potential-amplitude and subjective flicker threshold [18-20]. A study as recent as 2001 using square wave stimulation in vision also showed no particular change in the evoked potentials over the STZ [21]. Additional information on the limitations of SS as a measure of effects of stimulus repetition-rate is in the Discussion.

### QSD avoids the limitations of SS

The limitations imposed on SS studies by a uniform repetition-rate are avoided in QSD [14]. As we will show, QSD, using a small jitter of the SI, permits recovery of the brain's time-domain transient activity in response to rapidly-repeated stimuli, *even when the evoked-responses are overlapped in time*. This is possible because when there is jitter, the resulting temporal convolution of:

1) the **timing **of the jittered SI pattern, and

2) the brain's transient response is not the uniform, identically-repeated waveform of SS. The small differences that occur in the average allows recovery of the brain's response by deconvolution of the average by the **timing **of the jittered SI pattern [14]. The computational methodology for this process is called QSD (q-Sequence Deconvolution), and has been described in detail [14].

### QSD, in brief

Because QSD will not be familiar to the reader, we provide here a brief overview of the method for those who wonder how we can now record what was previously unobservable. Details specific to the results are in the Methods Section, and further descriptions are in the original QSD paper [14].

**Figure 1:**
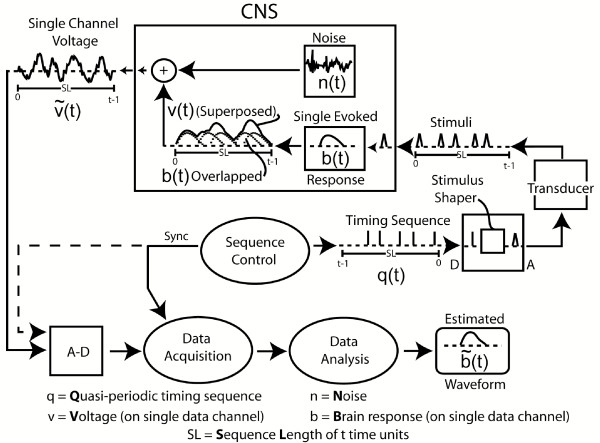
Diagram of QSD process. (Previously published [14].)

A diagram of the QSD process is shown in Fig. 1. To provide a jittered sequence of SIs, the "Sequence Control" unit (Fig. 1) outputs a binary timing sequence (*q(t)*) that consists ***solely ****of one's and zero's*. At the time of each "one", a stimulus-waveform generator activates a transducer that creates a stimulus, such as a click, flash, or electrical pulse. The result is a sequence of stimuli whose ***timing ****is determined by the timing sequence*. The *other stimulus parameters*, such as intensity, are *the same for ****every ****stimulus*. Each stimulus creates a single evoked-response (*b(t)*), but these responses overlap because the SIs (Stimulus Intervals, **start **-to-start) of the timing sequence are shorter than the duration of the evoked-response. It is mathematically proven in the QSD paper [14], that the process of superposing these overlapped waveforms is equivalent to a convolution of *b(t) *and *q(t)*, if, and only if *q(t) *is binary. The consequence is that if the binary timing-pattern of q(t) carries through to (*t*), *then b(t) can be "estimated" by deconvolution*. The brain response *b(t) *cannot be fully recovered because there is always some noise contamination, so the *estimated *brain response is expressed as  (*t*).

The mathematics of QSD can be expressed in a few equations. The following equation states what is illustrated in Fig. 1:

*(t) *= *[b(t) ^© ^q(t)] *+ *n(t) *    [Eq1]

that is, the recording on a channel ((*t*)) is the combination of the brain's evoked-response (*b(t)*) convolved with the timing sequence (*q(t)*), and that result is algebraically summed with the noise (*n(t)*). ***Note ****that the ****noise ****is ****not ****convolved*. Note also that all elements (*b,q,n*) have the same duration (*(t)*), which must be sufficiently long that *b(t) *has returned to baseline within the length of time of the q-sequence (*i.e*., within the SL = Sequence Length) [14].

In Eq1, the ^© ^symbol is used to denote the time-domain circular convolution. The recorded response (*t*) is a circular vector because it has been averaged on a 100% duty cycle synchronized with the cyclic, continuous, circular vector *q(t)*. Such circular vectors can be directly converted to the frequency-domain *without windowing*. Thus, Eq1 becomes, in the frequency-domain:

*(f) *= *[B(f)·Q(f)] *+ *N(f) *    [Eq2]

Note that the time-domain convolution function is, in the frequency-domain, complex multiplication. We can then recover the ***estimated ****brain response *in the frequency-domain [(*f*)] by dividing by the frequency-domain equivalent of *q(t) *(which is *Q(f)*), as shown by the Fundamental Equation of QSD:



As can be seen by Eq3, (*f*) can be recovered in the passband *if *the *Q(f)*'s in the numerator and denominator *are equal*. However, there must also be some noise contamination in (*f*) (consisting of *N(f)/Q(f)*). The estimated time-domain brain waveform  (*t*) is visualized by returning the frequency-domain values of (*f*) to the time-domain by an Inverse Discrete Fourier Transform.

Animated illustrations of the differences between QSD, "SS Responses", and standard averaging will be shown in the Discussion..

### "Early" results with QSD: Auditory Brainstem Responses

**Figure 2:**
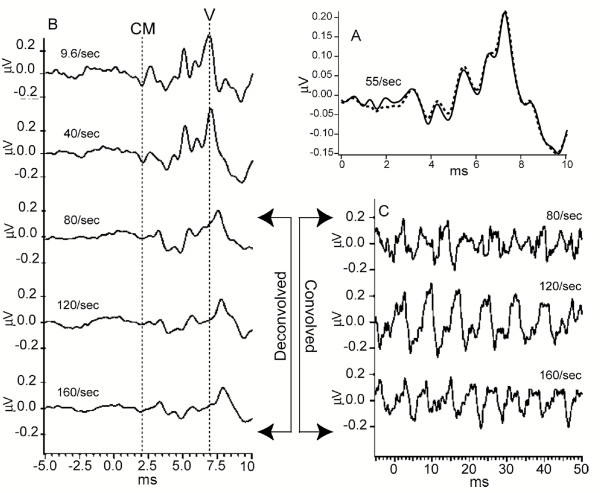
ABR recordings from two subjects. Taken from original paper [14]. Click stimuli delivered monaurally by Etymotic ER-2 insert-earphone at time '0' so that the stimuli arrive at the eardrum 1 ms later, due to tube-delay. **A: **ABR from male subject, clicks at 60 dbSL, at 55 S/s using a jittered sequence. The smallest SI in the sequence was 16 ms. The waveform found by QSD is the solid line. The dotted line is the 10 ms duration 'standard' average of the same data, triggered on each stimulus (no QSD). The similarity of waveforms shows that QSD returns the same waveform in a direct comparison (when there is no overlap). The passband was 120 to 2500 Hz. **B: **Recordings from female subject, clicks intensity 65 dbSL (relative to threshold measured at slowest rate). Passband filtered from 120 to 2000 Hz during deconvolution. At 9.6 S/s and 40 S/s waveforms obtained by standard averaging, one stimulus per sweep. Other traces obtained via QSD. Vertical dashed lines mark: (1) the timing of peak of the negative-going onset of the cochlear microphonic (CM) and (2) the peak of wave V. Note that the onset CM does not change latency with change in repetition-rate, but wave V does. **C: **The first part of the overlapped data from which the respective recordings in B were deconvolved (different time-scale). Note that absence of any 6 ms long flat portions in the convolved data, as compared with the pre-stimulus baseline in the deconvolved waveforms on the left.

Some QSD-derived waveforms that have already been published are needed in interpreting our A-wave Results. We first show the QSD-derived waveforms for the ABR (Auditory Brainstem Response). Figure 2 is taken from the original paper on QSD [14]; it shows first that plain averaging and QSD give the same results on the same data (recorded directly to the hard disk) [Fig. 2A]. In Fig. 2B are shown ABRs taken at 5 different stimulus repetition-rates. The two lowest rates (9.6 S/s and 40 S/s) were averaged with uniform SIs (standard technique). The remaining responses were obtained from jittered timing-sequences. Note that good waveform detail is possible, even at high rates. The negative-going onset of the cochlear microphonic has the same latency in all recordings (left-hand vertical dashed line). At 80 S/s and above, there is a shift in Wave V latency (right-hand vertical dashed line) and a reduced amplitude which may be due to a change in apparent loudness if there was sustained contraction of the middle ear muscles to the faster rates [22]. Between the cochlear microphonic and Wave V, most of the other ABR waves can be seen at the three highest repetition-rates.

The relative uniformity of the waveforms at different repetition-rates in Fig. 2B is in contrast to the averaged, convolved (superposed) data shown in Fig. 2C. Note that the superposed data traces of Fig. 2C are "quasi-Steady-State" responses, *i.e.*, they would be "Steady-State Responses" if there were no jitter. Note further that the peak-to-peak magnitudes of the convolved waveforms of Fig. 2C are *not proportional *to the corresponding peak-to-peak magnitudes of the deconvolved waveforms (Fig. 2B). For example, at the 120 S/s repetition-rate the convolved waveform has the highest peak-to-peak magnitude, but the deconvolved waveform at that rate is similar in magnitude to those of adjacent repetition-rates. This is one example that *between-rate differences *in "steady-state" responses *may not reflect ****actual ****brain-response differences*. (See also Discussion.)

There are several reasons to think that the waveforms of Fig. 2B are accurate. First, the direct comparison of QSD with standard averaging in Fig. 2A is good. Second, the waveforms at 80 S/s and above are all similar, despite the fact they are from different runs and that a different timing-sequence was used for each run. Third, the differences in waveshape compared with the slower rates are physiologically reasonable, showing systematic latency and amplitude changes. Fourth, there is one part of the waveform whose shape should be predictable: the pre-stimulus baseline should be relatively flat, as it is in the deconvolved waveforms (Fig. 2B). Note that there are *no comparable flat portions *of 6 ms duration in the convolved data of Fig. 2C (which has the same vertical scale as 2B).

### Initial results with QSD: G-waves

**Figure 3:**
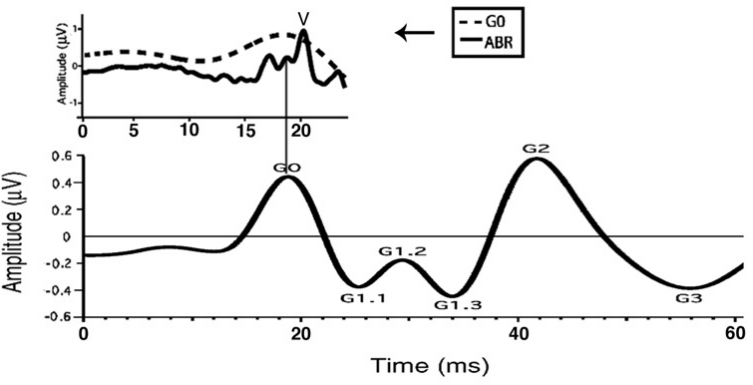
G-wave auditory evoked-response recordings. Modified figure from [69]. Recorded from one electrode pair: C3 to right earlobe. **Above: **G0 peak of G-waves (solid line; passband 30–120 Hz) compared with the ABR (dashed line; passband 120–3000 Hz). **Below: **G-waves, on the same time scale as in **A**, but with a *different vertical scale*. Note: The peak of G0 corresponds to the middle of the ABR.

Because of the relevance to interpretation of A-waves, we show G-wave data adapted from a previously-published figure. The waves in the 10–100 ms latency-range after an auditory stimulus are called the AMLR (Auditory Middle Latency Response) [23]. QSD-derived auditory evoked-responses (which we call "G-waves") are found within the "AMLR-range", using a passband of 30–120 Hz. Fig. 3 has been adapted from our first paper on these responses in which G-waves obtained from tone-pip stimulation at 40 S/s are shown.

Since the stimuli occur every 25 ms at 40 S/s, it is clear that the 80 ms-long response in Fig. 3 was overlapped in the averages before deconvolution (not shown). The upper trace of Fig. 3 shows that G0 is the "filter-integrated" ABR that is observed when G-waves are recorded with a 30–120 Hz passband. Fig. 3 shows that both brainstem and higher neural levels can be recorded in the same sweep. It will be apparent that this panoptical view also occurs with A-waves (see Results).

The letter "G" can serve as a mnemonic for "gamma" since the period of the G-waves is within the gamma-range of the EEG. We label these "G-waves", rather than the AMLR, because they were obtained at a stimulus repetition-rate which caused the responses to be overlapped. We define the term "G-wave" to be any *auditory *evoked-response within a latency range of 10 ms to about 100–125 ms (assuming the 30 – 120 Hz passband). It will be seen in Results that when the highpass filter has a lower value, the waves continue on for considerably longer.

## Results

### Overview of results section

We present our preliminary data on A-wave human evoked-response waveforms. We show visA-waves, audA-waves, and somA-waves, and find both differences and similarities in waveform as a function of stimulus repetition-rate. We also offer evidence that these unusual waveforms are not artifacts of the QSD calculations.

We first show the effect of stimulus repetition-rate on visA-waves. There are systematic differences as a function of repetition-rate (especially above and below STZ). We next show audA-waves, including examples of the variation of these waveforms, within day, and between days. While we do not have enough data for statistical analysis, there is evidence that the differences as a function of repetition-rate are not due to "selective data selection" by the authors.

In the next section we show that visA-waveforms are similar in shape to known "after-discharge" visual responses, even though visA-waves are obtained with continuous stimulation at high repetition-rates. We then show that differences in somatosensory somA-waves are seen above and below STZ.

To assuage worries that these new phenomena are artifacts, we then show the evidence we have so far accumulated that these responses are not generated by the QSD method, and hence need to be seriously considered as a new measure of brain activity.

### Introductory remarks

Using the QSD method with a filter passband where the highpass is below 30 Hz, we have found oscillatory waves some of which have periods *in the "alpha" range of the EEG*. The first author could not resist naming these "A-waves". A-waves are operationally defined as those waveforms obtained with a highpass less than 120 Hz, that have a duration longer than the SI used to evoke them, *i.e.*, the stimulus repetition-rate is fast enough that the responses overlap. The definition of "A-waves" does not require that the waveform have oscillations with a period within the alpha-EEG range, although many A-waves at supraSTZ rates have had such oscillations. A-waves without sustained oscillations have been recorded in response to subSTZ stimulus repetition-rates. Note again that the term "A-waves" *only *implies that the responses are longer than the SI, and does *not *require that the response have sustained oscillatory components, though many do.

### Effects of stimulus repetition-rate on visA-waves

**Figure 4:**
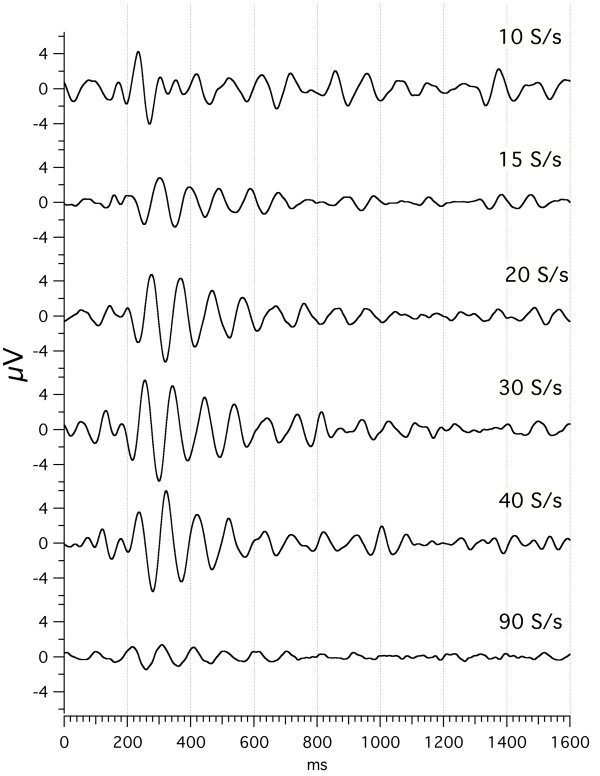
visA-waves recorded to a flash to the left visual hemifield, at various rates of stimulation. Sequence length = 1600 ms. Subj = Cg. Vertical scale = 4 V. Passband = 8–50 Hz. To see the *convolved, averaged data *from which this data was deconvolved follow this link: [see Additional file 6].

In Fig. 4, we show visA-wave responses at different flash repetition-rates. The data are shown with the full sequence-length of 1600 ms, which was the length of the circular vector before deconvolution. The convolved averages from which these waveforms were obtained can be seen in the figure which can be brought up from the Figure Legend of Fig. 4. These convolved averages indicate why these waveforms have not previously been observed.

**Figure 5:**
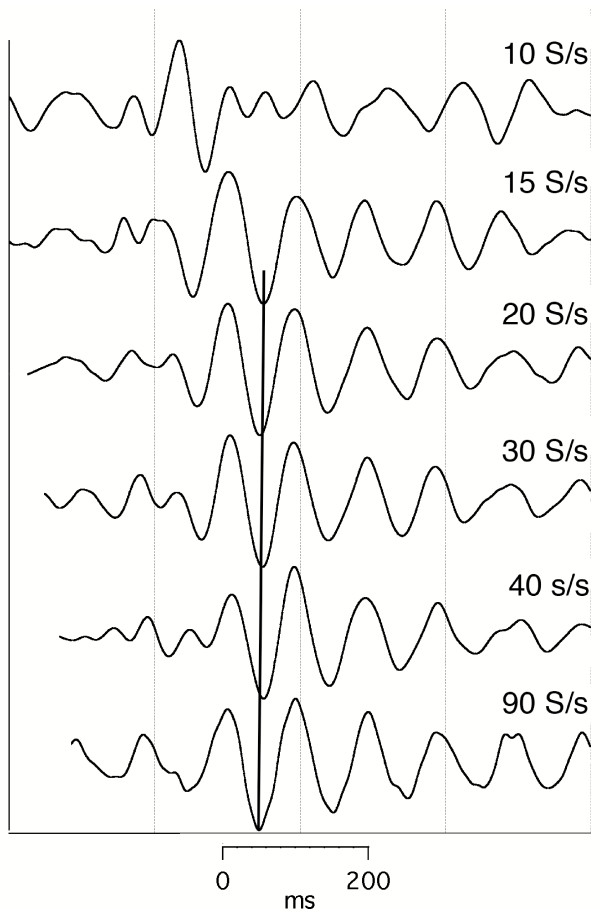
Same as Fig. 4, re-graphed with different vertical scales and with added latencies.

Returning to Fig. 4, there are systematic latency shifts that are not easily seen in the figure, so in Fig. 5 we re-plot the data. Fig. 5 differs from Fig. 4 in several ways:

1) Only the first 800 ms after the stimulus are shown.

2) The waveforms are *normalized *to an equal height by using different vertical scales on the traces.

3) The waveforms of 20 S/s through 90 S/s have been *moved *to the *right*, so that the second negative valley will align with the same valley in the 15 S/s waveform, as shown by the solid vertical line. The 10 S/s and 15 S/s waveforms have **not **been moved.

(The choice of the second negative valley was somewhat arbitrary, being chosen because the wave is large, present in all of the traces, and seemed to be the onset of consistent oscillations following it.)

*Note the considerable similarity in shape of the visA-waveform *across the rates at and above 15 S/s, though the amplitudes do vary (refer back to Fig. 4). The shift in latency necessary to bring about the alignment can be seen by the blank space at the start of the traces that were moved. The shift means that wave-peak latency *shortens *as the repetition-rate *increases*. Note also that *the waveform at the rate of 10 S/s ****was different ****from that seen at all the other rates*. We were surprised that even at a repetition-rate as slow as 10 S/s the response was longer than the SI, thus requiring QSD to obtain this response. Another surprise was finding such long-duration waveforms correlated to stimuli being delivered at such high rates. The amount of overlap can be seen in the convolved averages, which is accessed from the Fig. 4 legend.

**Figure 6:**
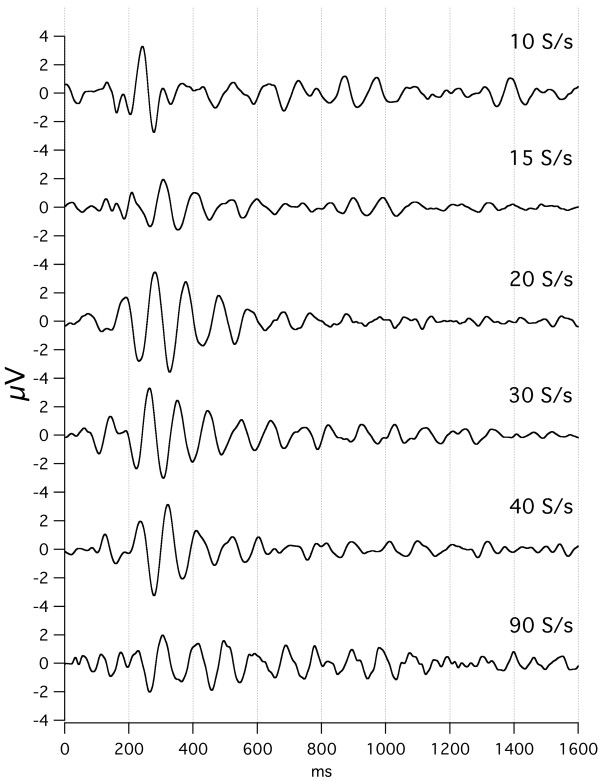
visA-waves recorded to a flash to the right visual hemifield, at various rates of stimulation. Sequence length = 1600 ms. Subj = Cg. Vertical scale = 4 V. To see the *convolved, averaged data *from which this data was deconvolved follow this link: [see Additional file 7].

**Figure 7:**
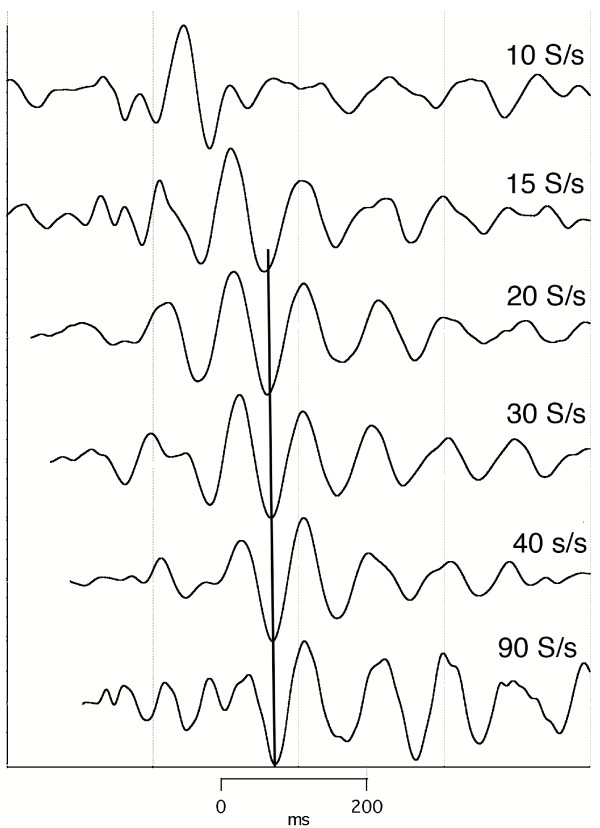
Same as Fig. 6, re-graphed with different vertical scales and with added latencies.

To show that the data presented could be replicated in the same subject, we show runs taken when stimulating the opposite visual field (Figs. 6, 7).

### Effect of stimulus repetition-rate on audA-waves

**Figure 8:**
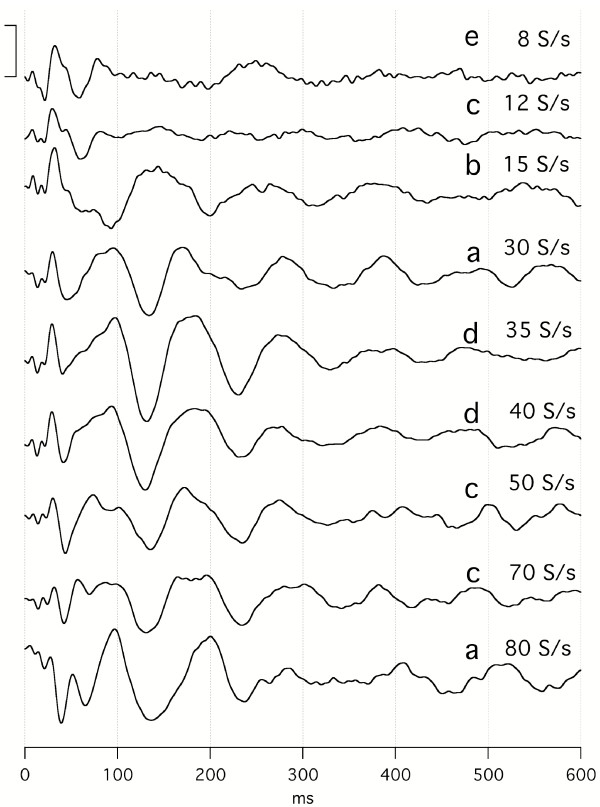
audA-waves over a range of stimulus repetition-rates, in a single subject (Ap). Full data 1600 ms long; only first 300 ms shown. Monaural right stimulation at 65 dBSL. Abscissa, ms; ordinate bar = 1 V. Filter 8–50 Hz. On the right, the letters a-e refer to the dates on which the data were taken. The number of days between recordings are as follows: a-b, 8; b-c, 85; c-d, 7; d-e, 27. To see the *convolved, averaged data *from which this data was deconvolved follow this link: [see Additional file 8].

**Figure 9:**
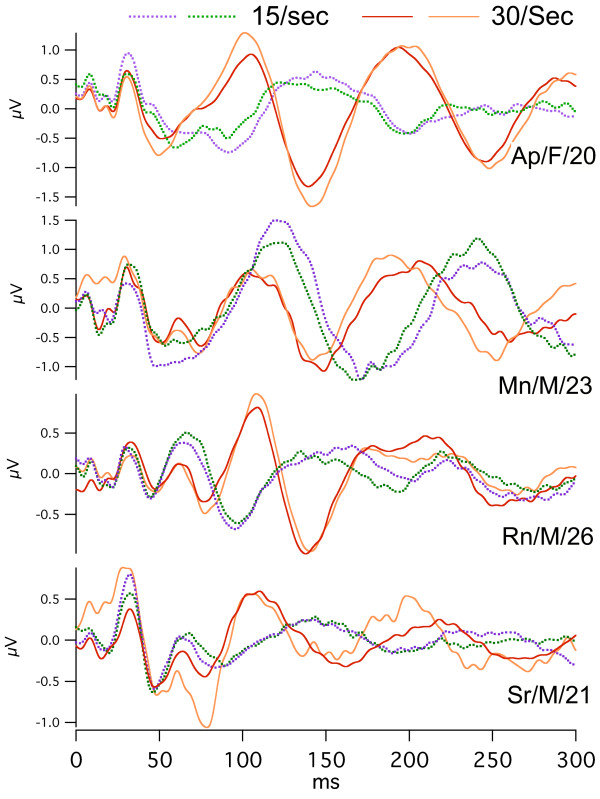
audA-waves from four subjects. Subject identifiers = code/gender/age. Recorded from the C3'-O2 channel, at two repetition-rates: 15 S/s (dotted lines) and 30 S/s (solid lines), with overlapping of *replicate runs at each rate*. The jitter maximum was 12% around the mean. Monaural, right ear, Etymotic tubephone stimulation. Abscissa, ms; ordinate V; Filter: 5–120 Hz. Averaged data before deconvolution 1600 ms long; only the first 500 ms are shown. *Note *that the vertical scales differ between subjects.

The effect of stimulus repetition-rate on audA-waves is shown in Fig. 8, with repeated runs from subject Ap. Again, the *convolved averages *("raw data") can be accessed from the Figure Legend. The data of Fig. 8 was taken over a large number of days because each trace required a 40 min run. From 30 S/s to 80 S/s the A-wave oscillations (that start at a latency of about 80–100 ms) are quite similar *despite the differences in repetition-rates*. On the other hand, the audA-waveforms from stimulation at 8 S/s to 15 S/s are smaller *and *appear to have an opposite polarity at both 130 ms and 230 ms. The waveform at 15 S/s is unique in all of the A-waves, in being different from waveforms *both *above and below it in repetition-rate. We puzzle whether this is very close to the "fusion-boundary" of 18 S/s, mentioned in Background relative to early work in fusion. (More comparisons of waveforms above and below the auditory STZ will be shown in Figs. 9 and 15.) It is notable that the *visA-wave negativity *in the range of 260–360 ms in Fig. 4 shows shortening of peak-latency as repetition-rate increases, whereas the *audA-wave negativity *at about 130 ms in Fig. 8 does not change peak-latency with repetition-rate.

### Variation in audA-waves, within-day, between-day

In Fig. 9 we show audA-wave run-to-run differences in 4 young-adult subjects, 3 male, and one female, at two repetition-rates (below and above STZ). In Fig. 9, note the similarities of the waveforms between rates (within a subject) up to about 50 ms, with marked divergence thereafter. G-waves are visible as the overlapped waves before 50 ms in Fig. 9. G-waves are much less affected than are the A-waves by *both *the repetition-rate differences *and *the run-to-run differences. We tentatively consider the latency interval between 50 and 80 ms to be the transition between G-waves and *oscillatory *A-waves in auditory responses.

We hypothesize that the variation before 50 ms is due to the low-frequency EEG "noise" within the A-wave passband. (When we have studied auditory G-waves with a passband of 30–120 Hz, this degree of variability is not present. Hence, the EEG contribution to the waveform after 50 ms is presumably about the same as the variation before 50 ms.) The A-wave *oscillations *start at a latency about 80 ms. A notable feature in Fig. 9 is the clear difference between the waveforms of 15 S/s and 30 S/s after about 50 ms. These differences are *notably larger *than *the run-to-run differences*. So, we consider that the *repetition-rate differences *are ***not likely to be ***due to *run-to-run differences*, despite the absence of calculated statistical probabilities. (The data was collected in exploratory fashion without the predesigned form required for the rigorous statistical analysis that we hope to provide in later papers.)

Although in Fig. 9 we show only the first 300 ms of these audA-waves, in 2 of the 4 subjects the audA-waves continued past 500 ms (not shown). Consequently, the original (convolved) data is highly overlapped, as was shown with respect to the audA-waves of Fig. 8 (see Legend). The first positive peaks at 30 S/s in Fig. 9 are about 93 ms apart and the first negative peaks are about 110 ms apart. These two periods would correspond to about 11 Hz and 9 Hz respectively, placing them *within the "alpha-frequency" range of the EEG*.

The correspondence between audA-wave peaks and the peaks of traditional AEP waves is unclear at this point. As is known from the ABR, if experimental conditions are changed, the waveform at a fixed latency after the stimulus can be due to different neural generators. Clearly *changing the stimulus repetition-rate is a changed experimental condition *that may also change the neuronal contributions to observed peaks. So, we choose, at this time, not to use previous peak-naming conventions based solely on latency.

**Figure 10:**
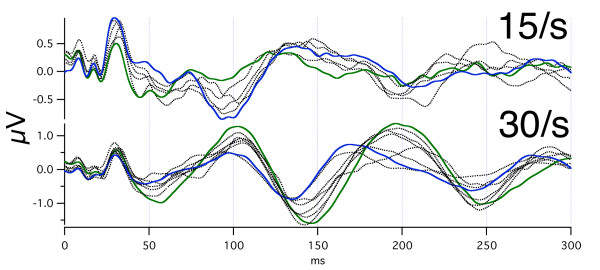
Day-to-day differences in audA-waves at two different repetition-rates in subject Ap. Monaural stimulation, right ear. The recordings were first taken over 15 days, and then 3 months later were taken over 42 days. The 15 S/s data shows 12 overlapped traces/days, and the 30 S/s data shows 9 traces/days. All traces are dotted, with the exception of the two traces having a maximum or minimum at 100 ms (to show how the same trace differs at other latencies). Note that *despite the day-to-day variation*, the polarities are opposite at about 100 ms, about 140 ms, about 200 ms, and about 250 ms.

Returning to the issue of audA-wave variability, Fig. 10 shows the *between-day run-to-run variation *seen in female subject Ap (same subject as in top trace of Fig. 9, and in Fig. 8). Note that in Fig. 10 the 15 S/s and 30 S/s waveforms are shown on two different vertical scales. The between-day run-to-run variation is greater than the *within-day *run-to-run variation (top trace of Fig. 9). Especially important is the fact that *even the waveforms at the extremes show the rate differences*. That is, the waveform-differences due to repetition-rate are larger than any between-day differences, which argues against the idea that the differences shown in Fig. 8 are due solely to investigator-selection of data.

### Somatosensory responses (somA-waves)

**Figure 11:**
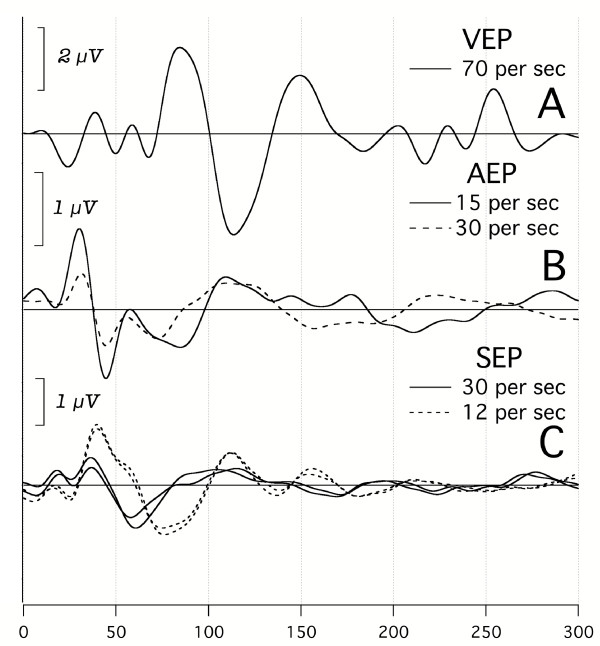
Somatosensory A-waves (somA-waves) compared with visA-waves and audA-waves in the same subject. **A: **A single visA-wave run, stimulating the left hemifield. **B: **audA-waves at two different rates. Monoaural stimulation in right ear, Dau-chirps at 45 dBSL. **C: **somA-waves from right median nerve stimulation sufficiently strong to cause thenar muscle contraction. Replicate runs are shown at two different rates. *Note: this male subject was 74 yrs old, and had some high-frequency hearing loss*.

Having found a distinction between subSTZ and supraSTZ waveforms in the auditory and visual systems, predicting that they might also be found in the remaining cerebral cortex sensory system was irresistible. In Fig. 11C we show our only recordings from electrical stimulation of the somatosensory system. The waveform and latency differ for the two recordings, at 12 S/s and 30 S/s. We did not obtain the other recordings, above and below these rates, that would be necessary to prove that these differences occur only with a transition at the STZ. But the waveforms are consistent with this being the case.

It is unclear whether persistent oscillations in somA-waves will be obtained in future recordings, since they do not occur in Fig. 11C. It is notable that in this 74 yr old male subject, the visA-waves (Fig. 11A) and audA-waves (Fig. 11B) also recorded do not show the prolonged oscillations, either. Thus, the absence of oscillations in somA-waves may be a function of the age of the subject, or some other factor.

### Similarities between A-waveforms and "after-discharge"

**Figure 12:**
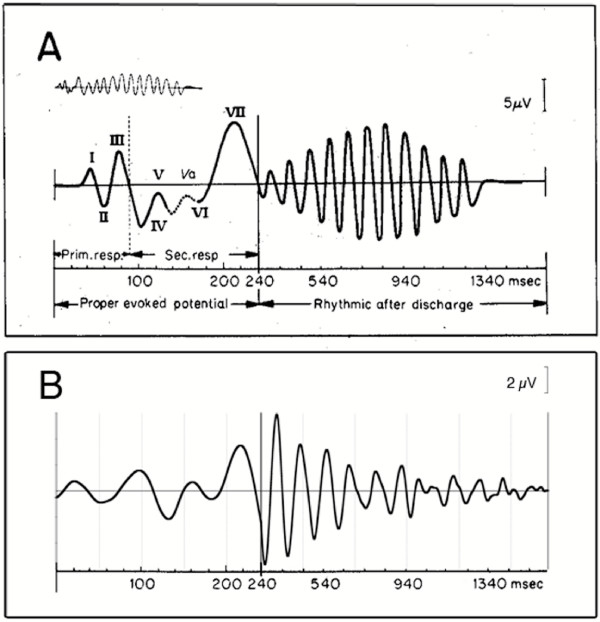
Comparison of a published "afterpotential" waveform and a visA-waveform, on two different time scales. **A: **"Classic" afterpotential, as shown on pg.379 of Regan's book [70], originally from Ciganek [71]. **B: **A visA-wave taken at 30 S/s. This is the same as shown in Fig. 4 but is plotted on the two different time scales of **A**.

Reasonable doubts about the validity of a new waveform can be assuaged, not only by showing that they are not artifacts (next Section), but also by comparison with prior research results. While no recordings have ever shown A-waveforms at the stimulus repetition-rates we use, prolonged "after-discharge" has been previously observed in the visual system. A "textbook" figure of after-discharge is shown in Fig. 12. Note that in Fig. 12 the time axis has two different scales. We will discuss each in turn.

The first 240 ms shows the early waves in the VEP. Some of our visA-wave recordings show them and some do not (see Figs. 4 and 6); we do not understand why. The similarity between Fig. 12A and 12B suggests that we are recording the early events in the VEP, *despite the overlap *(SI = 33 ms at 30 S/s).

We now turn to the oscillations with about a 110 ms period that are shown at the slower time axis in Fig. 12A, in the latency range of 240–1340 ms. The visA-waves of Figs. 4 and 6 also show such oscillations out to about 1000 ms. So, our QSD-derived visA-waves show activity to high-rate stimulation that has been previously known *only *with respect to ***slow rate ****stimulation*.

**Figure 13:**
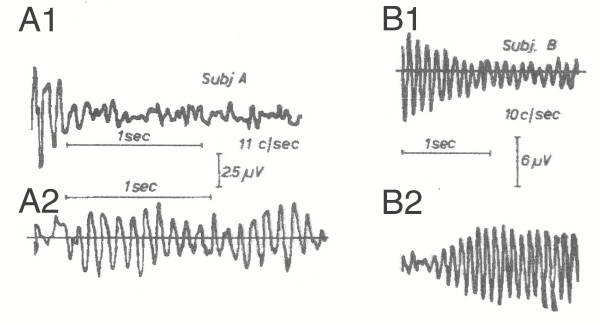
Responses from trains of sinusoidally-varying light with a modulation depth of 10%, at rates near that of alpha waves. (Copied from Tweel, et al. [17].) Recordings in two subjects, at 11 Hz for Subject A, and 10 Hz for Subject B. The upper traces show the decay of response at the end of the train. The lower traces show the response build-up at the start of the train. Subject B shows much longer build-up and decay than does Subject A, and larger waves as well.

Regarding afterdischarge activity from continuous stimulation *at a repetition-rate ****within the alpha range***, we show, from the literature [17], Fig. 13. The overall shape of the oscillations in the after-discharge waves in Fig. 13 are similar in overall shape to the oscillations in the visA-waves of Fig. 4 (especially Fig. 13A2). The major difference is that similarly-shaped visA-waves are found *at markedly different repetition-rates*, not just a repetition-rate near 10 S/s (which sums waveforms with a similar cyclic rate).

One might imagine the persistent waveform after the end of stimulation (A1 and B1 in Fig. 13) as being due to length of the individual responses and the decline as due to the diminishing amount of overlap. One can also imagine that, at the start of a train of stimuli at a rapid repetition-rate, there would be summation of the overlapping visAwaves of Fig. 4, which might have a shape similar to the "onset response" in Fig. 13B2. However, we know that at the beginning of a train of stimuli that the responses *cannot be identical*. *There must be ****a transition ***from the subSTZ waveform to the supraSTZ waveform *at the ****start ***of a prolonged stimulation at a (nearly) uniform repetition-rate. This is shown in the next Section.

### The morphing of subSTZ waveform to supraSTZ waveform

**Figure 14:**
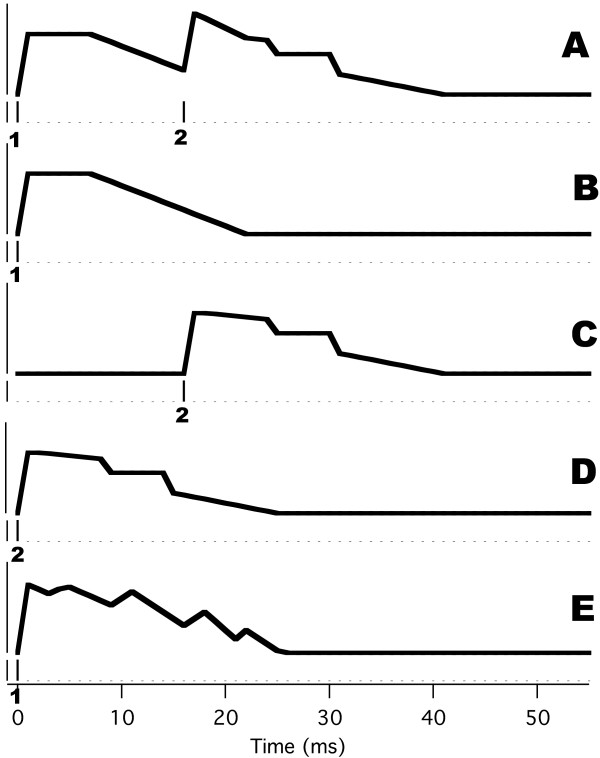
Diagram of *the method used *to compare A-waves. This is a diagram of the method used to compare: 1) the response to the second stimulus in paired-stimuli, and 2) the deconvolved response from QSD at the same SI. The goal is to determine the waveform at the start of a stimulus train, compared with the asymptotic response in the middle of the train. To make the comparison, the mean period in the QSD sequence is the same as the time between the two stimuli in the pair. **A: **The response to a pair of stimuli, where *the response *to the *second stimulus *of the pair *is different *from the response to the first stimulus. **B: **The response to a single stimulus. **C: B **subtracted from **A **gives just the response to just the second stimulus. **D: C **is moved to the left so as to ease the comparison with **A**. Note the time-scale has moved, but the time of stimulation for this response is now at the beginning of the sweep, as it is in **B**. **E: **The response to a single stimulus in a continuous stimulation at a repetition-rate with a period the same as in **A**. This waveform was deconvolved by QSD from overlapped data.

We now show that the audA-waveform at the *start *of a sustained train of stimuli is *not *the audA-waveform produced *during *sustained stimulation. The experiment was conducted by analysis of three runs of data, as *diagrammed *in Fig. 14:

1) A run is recorded with slowly repeated pairs of stimuli (the time between stimuli inthe pair being short, but the time from start-of-pair to start-of-next-pair is long), where the *overlapped responses *are shown in Fig. 14A, along with the timing of the two stimuli, 1 & 2. To remove the overlap in the response to the pair we record the next run.

2) A run using single stimuli at a slow rate gives the *response *shown in Fig. 14B.

3) The response of the single-stimulus run (Fig. 14B) is subtracted from the response from the paired-stimuli run (Fig. 14A), giving the *response *to *just the second stimulus*, as shown in Fig. 14C.

4) The response to just the second stimulus (Fig. 14C) is then moved to the left (note stimulus mark in Fig. 14D), so as to permit easy comparison with other responses relative to the time of the stimulus that generates them.

5) A third response is obtained using high-rate stimulation, where the SI in the continuous stimulation is the same as the timing between the stimuli in the pair. The response would be deconvolved using QSD, to obtain the response to each stimulus (Fig. 14E).

6) Finally, *the three responses are compared:*

a) The response to a single stimulus (Fig. 14B), which must be the same as the response to the first stimulus in a stimulus train (if the repetition-rate is slow enough to be equivalent to "no prior stimuli").

b) The isolated response to the second stimulus (Fig. 14D), which is different because it was affected by the prior (first) stimulus.

c) The response to a *sustained repetition *of the stimulus (Fig. 14E), which is the "steady-state" response due to stimuli separated by that period (which is also the period between the pair of stimuli in Fig. 14A). This response must occur at some point in the stimulus train if the stimulus train is long enough.

**Figure 15:**
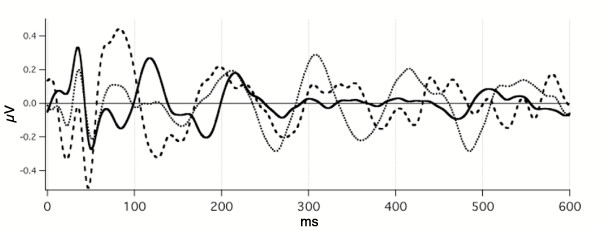
Demonstration that A-waves are not immediately generated by the first pair in the run. Abscissa, ms; ordinate V. **Solid trace: **The response to a single Dau-chirp presented at 15 S/s using a q- sequence. **Dotted trace: **The response to the second of a pair of Dau-chirps with the timing between the pair at 14 ms (the period of 70 S/s). The timing from start-of-pair to start-of-pair was 15 S/s, using the same q-sequence. See text for the method of extracting and shifting this waveform. **Dashed trace: **The response to the same Dau-chirps when they are presented in a jittered q-sequence, mean of 70 S/s. NOTE: The dotted trace is mid-way between the solid and dashed traces within the first 120 ms, i.e., the response to the second stimulus of the pair does not equal the response to continuous stimulation.

In Fig. 15 we show that audA-wave data shows that the waveshapes of the three waveforms described above as 6a, 6b, and 6c are *not the same*. Note the following in Fig. 15:

1) The *Solid *trace is the response to Dau-chirp stimuli jittered at a mean rate of 15 S/s (same sequence as used for timing the start-to-start of the pairs). The jittered SI was required because the audA-waves are longer than the SI.

2) The *Dotted *trace in Fig. 15 is the response to the second stimulus of a pair ofDau-chirps where the pair had a fixed interval of 14 ms from start-to-start. The inter-pair interval (start-to-start) was jittered around a mean interval of 67 ms (= 15 S/s), so that it was the same rate and pattern as for the response to the single stimulus (Solid trace). Because of the overlap of the first response to the second, it was necessary to do the subtraction, as diagrammed in Fig. 14C and then shift as in Fig. 14D.

3) The *Dashed *trace in Fig. 15 is the deconvolved response to a Dau-chirp, recorded with a stimulus repetition-rate of 70 S/s (which has the same period [14 ms] as the separation of the pairs (Dotted trace).

Clearly, Fig. 15 shows that a pair of stimuli are insufficient to evoke the audA-waveshape obtained from sustained rapid stimulation, although some "elements" of the sustained waveform begin to develop by the second stimulation. For example, note the opposite polarities of the two responses (solid and dashed lines) at about 90 ms and at 120 ms, with the dotted trace having intermediate values. Thus, the "full" audA-wave takes some number of repetitions of the stimulus before reaching an asymptotic waveshape. This finding has implications for the kinds of neuronal mechanisms that are involved in generation of A-waves, and also justifies our not trying to overlap visA-waves to mimic increasing or decreasing "after-discharges" as shown in Fig. 13.

### Evidence against artifactual waveforms

With any new technique, especially if it presents unusual results, it is reasonable to wonder whether artifacts are created of such magnitude as to produce the unexpected. We provide now a number of different lines of evidence against artifactual generation of waveforms by the QSD technique.

The first line of evidence is that runs can differ within a sensory system as a function of stimulus repetition-rate, as shown in Figs. 4, 6.

A second line of evidence is that very similar-appearing waveforms can be obtained from *different-appearing *convolved data, as shown in the convolved data used to find the waveforms of Fig. 8 (see Legend to access the convolved data file).

**Figure 16:**
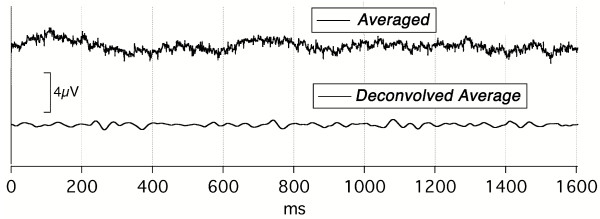
Control recordings when the flash was covered with cardboard. Vertical scale = 4 V. **Top trace: **Averaged EEG ***with no stimulus***. **Bottom trace: **The *deconvolved average *of the top trace ***(no response)***.

A third line of evidence involves deconvolution when there is no correlated brain activity, i.e., no evoked-response. When the visual stimulation was stopped by covering the flash unit with cardboard, an average of the EEG was obtained that was not influenced by the hidden flashes (Fig. 16, Top). (Recall from Fig. 1 and Eq2 that the EEG "noise" is *uncorrelated *with the timing of the stimuli.) *Deconvolution of the EEG average did not show any evoked-response *(Fig. 16, Bottom). Clearly the deconvolution calculation *per se *does not generate evoked-responses.

**Figure 17:**
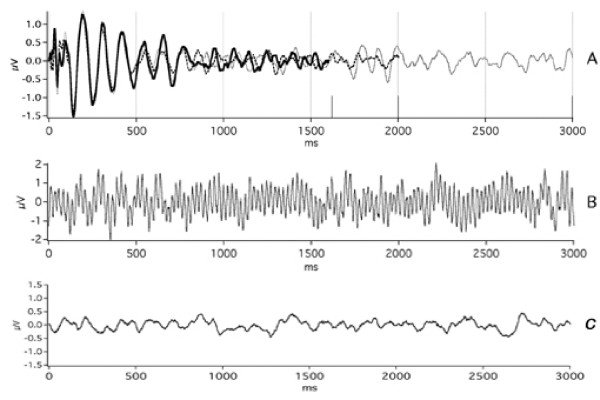
audA-wave data from subject Mn. Monaural right ear stimulation. Abscissa, ms; ordinate V; Filter: 5–130 Hz. Full data length shown. **Top trace: **audA-waves from stimulation at 40 S/s taken on three separate Sequence Lengths: 1.6 s, 2 s, 3 s. Note that up to about 500 ms the waveforms overlay with only small differences. From 500 to perhaps 1400 ms there is some agreement, but clearly there are more differences. **Middle trace: **Overlapped (convolved) data from which the 3 s waveform in the Top trace was deconvolved. There are 20 stimuli every 500 ms. **Bottom trace: **Control EEG obtained without stimulation, then averaged, and deconvolved. Note absence of any "response".

The fourth line of evidence is shown in Fig. 17; *similar audA-wave results are obtained with completely different q-sequences*, of different lengths, though each have the same mean repetition-rate: 40 S/s. One might expect an artifact to differ with different calculations. The Top trace of Fig. 17 shows the overlap of data from three different runs, each with a q-sequence of a different length: 1.6 s, 2.0 s, and 3.0 s. The between-run agreement is highest within the first 500 ms, and has reasonable agreement out past 1000 ms. Note also that the G-waves, though barely seen at the far left of the Top trace, also overlap. This was verified by expanding the trace (not shown). Note that the Top trace of Fig. 17*also shows the run-to-run variance in this data*, taken from a male subject with as many as 14 days between runs.

The time-domain average for the 3.0 s SL is shown in the Middle trace of Fig. 17. The periodicity-peaks with the shortest inter-peak time occur at the stimulus repetition-rate. No consistent 100 ms periodicity is seen in the Middle trace, in *marked *contrast to the deconvolved waveform in the Top trace. In the Bottom trace of Fig. 17, we shut off the auditory stimulation, and averaged for the same length of time as taken in recording the Middle trace. When that average was deconvolved, the result was the Bottom trace, which shows no evoked-response, providing further evidence to that shown in Fig. 16.

**Figure 18:**
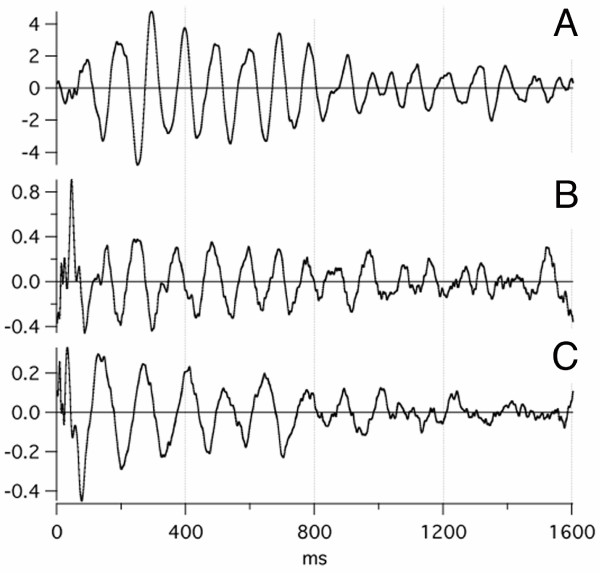
A-waves to either visual or auditory stimulation, using the *same *q-sequence. Abscissa: ms; ordinate V. Flash traces are inverted to correspond to VEP convention. **A: **visA-waves to flashes at 40 S/s. Male subject, Bt, 17 yrs. NOTE the ordinate – the visual responses are much larger than auditory responses. **B: **audA-waves to Dau-chirps at 40 S/s, *same timing sequence and same subject as in A*. NOTE that there are differences at short latencies (no G-waves in A), and in the duration of the A-wave oscillations. NOTE that the "jaggedness" of this trace may be due to the increased gain, as compared with A. **C**: audA-waves to Dau-chirps at 40 S/s, *same sequence *as in B but the subject is *different *(Male subject, Ma, 26 yrs).

A fifth line of evidence is the differences obtained between different sensory systems *using the ****same ****q-sequence*. As shown in Fig. 18, if the *same q-sequence *is used with recordings from the *same subject*, when recording visA-waves (Fig. 18A) or audA-waves (Fig. 18B), the A-waveforms are clearly different. This argues that the q-sequence calculation is not a major determinate of the waveforms. Furthermore, if the *same q-sequence *is used in recording a *different subject*, the audA-waves show both differences and similarities (compare Fig. 18C with 17B).

**Figure 19:**
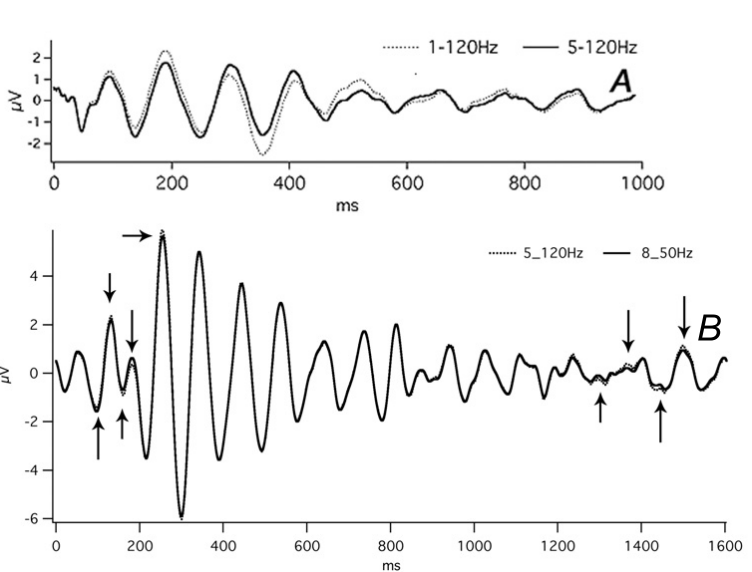
The effect of filtering on the overall shape of audA-waves and visA-waves. **A: **Subject = Mn. Monaural right ear stimulation at 40 S/s. Abscissa, ms; ordinate V. The sequence-length was 3 sec, of which only the first 1500 ms are shown. Run time = 100 min (1 hr, 40 min). **Dotted lines = **Data passband filtered 1–120 Hz. **Solid lines = **The *same data *filtered 5–120 Hz. (Note that this is the only recording shown in this paper that shows data with the highpass filter down to 1 Hz.) The effect of the filter (solid line) is to create a monotonic descent of the peak heights, which appears as a damped sinusoid, but that the brain's response (dotted line) actually has an increased positive peak just before 200 ms, and an increased negative valley at about 375 ms. The waves after about 475 ms have a magnitude within the noise level of the rest of the sweep (1000–3000 ms – not shown). *Note also *the filtered waveform (solid line) is more regular than the 1–120 Hz data (dotted line). **B: **Subject = Cg. Flash stimuli, left visual hemifield, 30 S/s. Same data as Fig. 12. **Dotted Lines = **Data passband filtered 5–120 Hz. **Solid Line = **The same data as the Dotted Line, but passband filtered 8–50 Hz. The differences due to the narrower passband are small – some are indicated by arrows.

One form of "artifact" can be distortion of the waveform by the stopbands of the filter. Each q-sequence requires the use of a passband filter depending upon the constraints used when searching for the sequence [14] The question naturally arises as to whether the 5–120 Hz passband distorts any part of the audA-waveform, or the 8–50 Hz passband distorts the visA-waveform. We show in Fig. 19A, that when the filter passband is 1–120 Hz, the audA-waves are more irregular in height than when *the same data *is filtered at 5–120 Hz. A-waves have the appearance in our other figures of a damped-sinusoid with a rather-uniform *monotonic amplitude reductio*n. Fig. 19A shows that the uniform amplitude reduction is *a mild filtering effect*. An additional, important aspect of Fig. 19A is that by having the filter passband wide-open (1–120 Hz) we show the *audA-waveshape *unaffected by "waveform selection by filter". Fig. 19B also shows that there are minor effects on visA-waves of the 8–50 Hz passband (see regions indicated by arrows), on data which was obtained with a more open filter (5–120 Hz). In the absence of a severe filter effect, waveforms shown that have been filtered either at 5–120 Hz or 8–50 Hz are the brain's responses, subject to the mild filtering mentioned above (and severe filtering of the ABR [the passband of which is usually 100–3000 Hz]).

## Discussion

A-waves, being a new evoked-response phenomenon, raise a number of issues, none of which can be definitively settled in an introductory paper such as this. Instead, we hope to indicate in this discussion what questions the findings generate, and in what ways these new phenomena might be useful.

### Trivial coincidence, or tantalizing clue?

**Figure 20:**
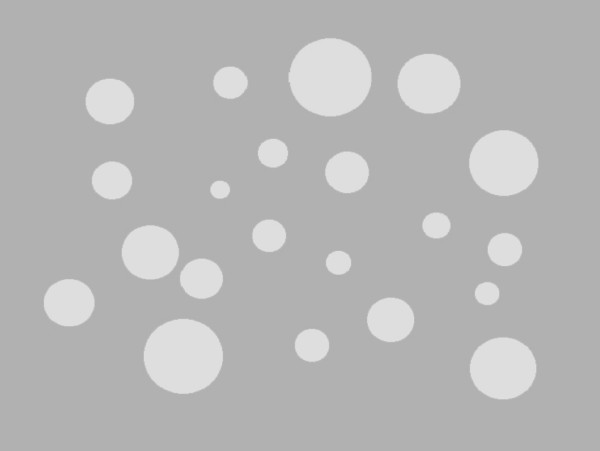
Demonstration of the effects of rate of visual stimulation on detection of image changes. **A: **Steady presentation of a field of light gray disks on a slightly darker background. **B: **Same as **A **except the disks are flashed at a rate of 2.4 S/s and *one of the disks is moving an amount equal to its radius*. [click "**MovieB**" below to see this]. **C: **Same video frames as in **B **except presented at a rate of 12 S/s (same as frame rate of movie) [click "**MovieC**" below to see this] **MovieB **[see Additional file 12] **MovieC **[see Additional file 5]

Mindful that "coincidence implies causality but does not prove it", the consistency of waveform differences in the visual, auditory, and somatosensory systems on either side of the STZ provides powerful motivation for producing some wide-ranging speculation. We demonstrate our primary speculation by means of Fig. 20, which shows a group of grey dots of different sizes. The presentation is steady (at the refresh rate of the screen you are watching). You are now going to see the same screen flashed, where one of the dots will move back and forth a distance of about its radius.

(Disclaimers: 1) Because of the characteristics of computer monitors,*we cannot duplicate the experimental conditions *that were used in our *visual *experiments. For example, we are limited to just the frame-rate for changes, and for the duration of/the stimulus. In our experiments the stimuli were brief, allowing manipulation of the SIs. 2) There are many factors that can influence this effect. For the purposes of this discussion, only the *rate effect *will be at issue.)

This demonstration is intended to provide you with the answer to the following question: ***Does the rate of presentation affect your ability to determine which dot moves***?

1) First go to Fig. 20 and then try MovieB (see Fig. 20 legend). The repetition- rate is 2.4 S/s with a 20% duty cycle.

2) Next try MovieC (see Fig. 20 legend). The repetition-rate for MovieC is 12 S/s with a 100% duty cycle. Hopefully you now answer the question in the affirmative, and that it is easier to see the dot move under the conditions of Button C. Note that seeing the dot move did not require any conscious effort, or any prior use of "attention". The detection of the moving dot is automatic (and presumably a relatively low-level of extraction of a changing stimulus embedded within a background that appears unchanging because of fusion).

The presence of this psychophysical phenomenon raises two questions:

1) What are the neurophysiological mechanisms that underly this psychophysical experience?

2) What functional role might such mechanisms play? Since we cannot immediately answer the first question, let's start with the second.

With regard to the "functional role" that this neurophysiological mechanism plays, a "scene-presentation" with most elements having a repetition rate above STZ provides a means to rapidly identify a *change in the visual field*. Whereas, when one is presented with the same stimuli at a subSTZ repetition-rate, it is very difficult to identify the dot that moves, even when one knows which dot to look at. *For ease of speculation about neurophysiological mechanisms*, let us assume that a "single" stimulation is followed by a single firing of the cells in the early part of the response (as is possible if the stimulus magnitude is adjusted to be moderate – neither near threshold nor near saturation – *and *the stimuli are brief). Certainly this kind of firing can be found at sensory cells in the PNS (Peripheral Nervous System). We assume, in this case, that the firing of subsequent post-synaptic cells in the CNS (Central Nervous System) is not at a slower rate than the rate at which the PNS cell is being driven.

*If the ****assumption ****of a one-to-one correspondence between stimulation and firing ****be granted***, then the stimulus repetition-rate is *also *the firing-rate of these early cells (PNS and CNS) in the response. In this way, a change in stimulus repetition-rate is equivalent to changing the intensity of a steady, continuous stimulus to the PNS. From this we can conceptualize the following hypothetical "rule": Any part of a sensory field that is firing at a uniform rate above the STZ is "Ground", whereas the parts of the sensory field that are the "Figure" have one or more of the following characteristics:

1) A firing rate below the STZ,

2) A firing rate, though above the STZ, that is changing. (Our attempts to define "Figure" and "Ground" have always lead to either obvious or subtle circular definitions; we therefore will purposely avoid rigor.)

The effect of Fig. 20 relies on the Ground being presented *above STZ, while the Figure is presented below STZ. If we imagine that the sensation of "fusion" involves *the detection of unchanging "sameness", then there must be a memory of the immediate past, and an estimate for the duration of that memory can be made from our experiments. Based on our data, we would *roughly *estimate the *longest *duration of this "fusion-memory" is about 80 ms for peripheral vision, and about 60 ms for the auditory system. (Based upon the work of Lalanne [2] and Brecher [3] the value would be 56 ms [the period of 18 Hz].) That is, we predict that any sensory inputs that are repeated at *shorter ****unchanging ****intervals *than some short interval, will give the "supraSTZ response", where the word "unchanging" implies "less than the just-noticeable-difference for that stimulus repetition-rate" (a criterion apparently met by a low-jitter QSD because there is a fusion effect despite the jitter).

**Figure 21:**
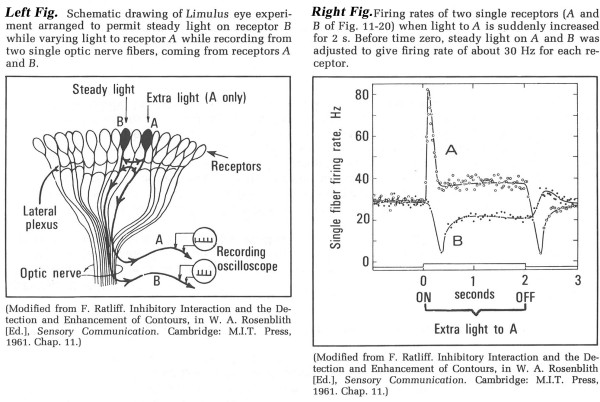
Limulus eye study, showing the effect of a step-increase in illumination to ommatidium "A". Modified from [72].

"Fusion-memory" needs be compared with the memory that occurs when the stimuli are separated by 150 ms or more (the number 150 is arbitrarily chosen to be larger than 100 ms, to avoid contentious arguments about alpha waves that may distract from this exposition). We will call this second memory "flash-memory" because the presentation that initiates it "comes and goes in a flash". Thus, brief *auditory *stimuli can also generate "flash-memory". A stimulus that is a step-function change probably generates a combination of flash-memory (transient) and fusion-memory (new steady-level), such as that shown in the firing rate for Cell "A" in Fig. 21R, relative to the step-increase in light. Thus, we hypothesize that the CNS response to the PNS activity indicated by Cell "A"'s firing rates will be different for the peak of differing spike-intervals at the onset of the step, as compared with the CNS response to the more uniform firing after adaptation to the new intensity. The CNS difference we imagine is that the changing firing rate corresponds to "Figure" whereas the more uniform rate corresponds to "Ground". Note that "Ground" takes some time to stabilize (after adaptation of the sensory ending's response to the step), which could correspond to the time for the subSTZ waveform to morph into the supraSTZ waveform.

When comparing the *quality *of "fusion-memory" with "flash-memory", fusion-memory is *much more accurate *for ***some aspects ***of the stimulus. For example, even though there is memory of a single flash of Fig. 20, it is not possible to remember enough detail to determine that one of the dots is changing position. The accuracy of fusion-memory is shown when something changes in an otherwise "stationary" scene. Look out a room-window at a scene in which nothing seems to be changing. A small movement of something in any part of the scene is rapidly noticed, even when the details of the scene are complex, unfamiliar, or even random. Somehow fusion-memory retains the "current-state" of the pattern of sensory-input, so that change is readily detected.

On the other hand, if change in a scene is sufficiently slow, it will go unnoticed – a neural phenomenon which is utilized by many predators who use slow approaches to prey, at a speed below that which triggers an "alerting" response in the sensory system of the prey.

To allow you to compare the accuracy of fusion-memory, with flash-memory, we offer a demonstration in the auditory system. For this demonstration, the sounds *must *be played through loud *speakers*, ***not****headphones*. If you have a stereo computer system, space the computer's speakers about 1.3 meters, or more, apart. Put one speaker at least 0.5 meter closer to you than the other, so that the "Dau-chirps" will appear to originate between the speakers, but closer to the near speaker (even though the "Dau-chirps" in both speakers will actually occur simultaneously). If you have difficulty observing the effects described, then try either moving the speakers a bit farther apart, placing yourself more asymmetrically relative to the speakers, and/or trying it in a smaller space, such as a closet. (The senior author has added the mention of the closet here, so that if someone finds you listening to buzzing sounds in a closet, you can produce *written evidence *to them that you are not *totally *crazy.)

To start stimulation at 2 S/s (uniform) use the following link [see Additional file 2] Adjust the intensity to be comfortably loud. Also set the audio-player to "loop" so that the sound plays continuously if it is not doing that.

Point your finger to the spatial location from which the sound seems to originate. Now rotate your head, left and right, over about a 60° range. Note that despite movements of the head relative to the speakers, the "location" of the sound is unchanged, and easily indicated by your pointing finger. Further, note that the subjective quality of the sound does *not change with this head movement*. Confirm the same observations by moving your head closer and farther from the speakers by about 15 cm. You have experienced what we call "flash-memory".

Now start stimulation at 100 S/s (uniform) using the following link [see Additional file 4]. Although the sound is raspy, a low-pitched tone is perceptible, in addition to higher-frequency timbre. Repeat the observations you made after pushing the 2 S/s button. Does the quality or loudness of the stimulus sensation change *with even small changes in head position or rotation*? (If so, return to "2 S/s (uniform).mov" to verify that you *cannot hear these differences at the slower rate*. [see Additional file 2]).

Is the accuracy of your locating the "source" the same as with the low rate, or has the "location" broadened? You have experienced what we call "fusion-memory".

(You might remember this the next time you encounter the sound of a solitary cricket's "chirp" and find it difficult to physically locate the cricket solely by its sound. The senior author presumes that the frequency of the cricket chirp is above your STZ, but somehow starts and stops without energizing flash-memory in predators (while having a different effects in other crickets). Another example is the lack of "location effect" for a sub-woofer in a multi-speaker sound system, where the sounds are cyclic repetitions that are supraSTZ.)

Did you notice that when you were listening to "100 S/s (uniform)" (fusion-memory) that you could hear the "glitch" when the sound-player on the computer reaches the end of the track and takes a moment to loop to the re-start? If not, try again: [see Additional file 4]. This is the *very feature *of the sensory input that fusion-memory is very good at detecting. Can you hear the glitch listening to "2 S/s (uniform)" (flash-memory)? The same timing "glitch" is there, too, but not detectable by flash-memory. You can verify this: [see Additional file 2].

*These effects are important in that the subjective differences observed can be hypothesized to be due to differences in memory functionality *between the shorter fusion-memory (at repetition-rates above STZ) and the longer flash-memory (at repetition-rates well below STZ). *We hypothesize that these psychophysical differences are due to differences in neural processing which are reflected in A-wave differences*. Another important aspect of these differences is that up to now *time-domain waveforms *from evoked-response research have been limited to those observable at subSTZ stimulus repetition-rates – *so the conclusions from such research only apply to flash-memory*. The issue of SS studies of supraSTZ rates is discussed later, in a separate section.

To demonstrate that the psychophysical effects are still present even though there is a small amount of jitter in the stimulus-intervals, as is required by QSD, we offer the same stimuli here, but with stimulus repetition-rates which are *jittered 12% as compared with the uniform rates*. "2 S/s (jitter)." [see Additional file 1] "100 S/s (jitter).mov" [see Additional file 3]

### Flicker-fusion and visA-waves

**Figure 22:**
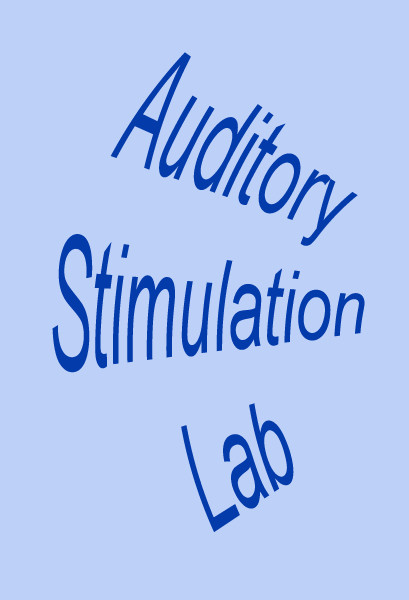
Sounds of different auditory stimuli, at different repetition-rates and at different percentage-jitters. The following files produce **clicks **that are **at uniform rate**, where the number is S/s. 2persec_click [see Additional file 17] 4persec_click [see Additional file 19] 6persec_click [see Additional file 21] 8persec_click [see Additional file 23] 10persec_click [see Additional file 25] 12persec_click [see Additional file 27] 14persec_click [see Additional file 29] 16persec_click [see Additional file 31] 18persec_click [see Additional file 33] 20persec_click [see Additional file 35] 22persec_click [see Additional file 37] 24persec_click [see Additional file 39] 26persec_click [see Additional file 41] 28persec_click [see Additional file 43] 30persec_click [see Additional file 45] 40persec_click [see Additional file 47] 50persec_click [see Additional file 49] 70persec_click [see Additional file 51] 90persec_click [see Additional file 53] 100persec_click [see Additional file 55] The following audio files produce **Dau-chirps **that are **at uniform rate**, where the number is S/s. **Same repetition-rates as for click's, above.** 2persec_dau [see Additional file 18] 4persec_dau [see Additional file 20] 6persec_dau [see Additional file 22] 8persec_dau [see Additional file 24] 10persec_dau [see Additional file 26] 12persec_dau [see Additional file 28] 14persec_dau [see Additional file 30] 16persec_dau [see Additional file 32] 18persec_dau [see Additional file 34] 20persec_dau [see Additional file 36] 22persec_dau [see Additional file 38] 24persec_dau [see Additional file 40] 26persec_dau [see Additional file 42] 28persec_dau [see Additional file 44] 30persec_dau [see Additional file 46] 40persec_dau [see Additional file 48] 50persec_dau [see Additional file 50] 70persec_dau [see Additional file 52] 90persec_dau [see Additional file 54] 100persec_dau [see Additional file 56] The following audio files show *the effect of increasing the amount of jitter*, using Dau-chirps at a mean rate of 40 S/s. The number indicates the percentage *jitter. The uniform 40 S/s is also provided for convenience, as the "No jitter – uniform" *file. The "MLS" Button is a Maximum-Length Sequence (= "m-sequence") of 511 stimuli, where *the minimum interval is 25 ms *(= 40 S/s). It is notable that as the jitter is increased, not only is the "tone" diminished, but the quality of the stimulus-sensation changes. We conjecture that a minimum number of consecutive SIs are needed before fusion-memory "locks in", and that larger *jitter prevents this*. "No jitter – uniform" [see Additional file 48] "12percent jitter" [see Additional file 58] "24percent jitter" [see Additional file 59] "36percent jitter" [see Additional file 60] "MLS" [see Additional file 57]

We have not done any formal testing to establish the relationship of A-waves to well-defined psychophysical phenomena. However, the region of stimulus repetition-rates *above and below which the A-waves show clear changes in waveform is the STZ, which in vision can be described without *much specificity as "where the flicker changes to fusion". When we tried, in a dark room, manipulating the flash-rate of a simple tachometer-flash system (no jitter), it was clear that there are *many *possible end-points that can be called "fusion". The central region of the visual field seemed to "go smooth" at lower frequencies than the peripheral vision which still could detect a flicker. There were moving "strings", "tendrils", or "webs" which ultimately "blended away", but at rates higher than that needed for fusion of central vision. For these reasons, we consider that there is no single "fusion" rate in our visual experience, and suspect that stimulus parameters, plus subject variables (such as accommodation and possible hysteresis) are likely to lead to different endpoints. Although we cannot provide this experience via computer monitors, we offer audio demonstrations in Fig. 22 for listening to sounds at different repetition-rates, with either "clicks" or Dau-chirps. These files are accessed via the Figure Legend of Fig. 22.

**Figure 23:**
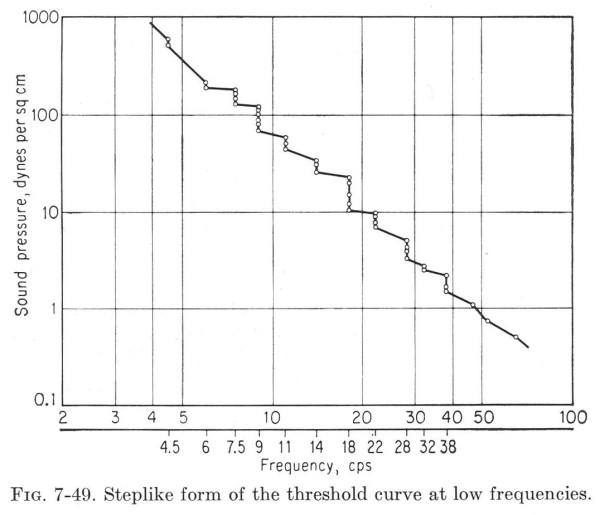
The quantal nature of frequency in auditory fusion. This is Fig. 7-49, on p. 260, of v. Bekesy's book [12].

Also in Fig. 22, we provide some sequences with increased jitter, not used in our experiments, to show the psychophysical effects of increased jitter. Note that the sounds of the non-jittered (uniform) 40 S/s [see Additional file 48] tend to form a low-frequency tone. That tone is less in the 12% jitter that we used [see Additional file 58]. At higher jitters the tone is gone – 24% [see Additional file 59] and 36% [see Additional file 60]. It is also missing with the MLS sequence [see Additional file 57]. This observation suggests to us that we were lucky to have not started with a larger jitter, and that future A-wave research needs to verify whether the waveforms differ at percentage jitters less than 12%.

We did not try to find an A-waveform "at *the *fusion-point" in our studies because such an endpoint might be highly variable, could differ with intensity, and, like threshold measurements, could involve many long runs. Our choices of stimulus repetition-rates were based upon guesses in the hopes of staying on either side of the STZ. It may be that the unusual audA-waveform at 15 S/s in Fig. 8 is within the STZ since it is unlike the waveforms at rates either *above or below it*. *There may well be interesting changes occurring within the range of about *12–20 S/s, as indicated by Fig. 23. In 1936, v. Bekesy reversed the usual procedure and kept the stimulus magnitude constant while varying frequency while searching for a fusion threshold, using a closed ear-canal stimulator [13]. He had this to say about fusion threshold:

"Careful examination revealed that the auditory threshold for low tones reflects the quantal character of neural processes. Thus if the frequency of the alternating pressures was changed slowly, *and without any variation of magnitude *[emphasis added], from 2 to about 50 cps, it was possible to observe that the loudness and the pitch did not vary continuously, but were altered in a stepwise manner.

"This discontinuity was most clearly perceptible in the region of 18 cps. As the higher frequencies were approached, there appeared a sudden increase in loudness, corresponding approximately to a doubling of the sound pressure. At the same time there was a doubling of pitch; the number of pulses, which were separately perceptible below 18 cps, suddenly became doubled, and the whole sensation became fused and acquired a tonal character (Brecher) [3]. This tone was still extremely rough, and the roughness gradually declined as the frequency was raised further. This frequency therefore can properly be designated as the threshold of fusion (Brecher) [3]. It is practically the same for all the sensory modalities..."

For further work related to the quantal nature of this and other data, see Geissler [24]. For a review, see Kompass [1].

We might expect that A-waves might be affected by these factors, although the A-waves we have shown are all recorded "above threshold" in terms of intensity and repetition-rate.

The relevance of QSD to psychophysical research on the phenomenon of "fusion" is that QSD provides a means of correlating observable brain activity with psychophysical endpoints (if the endpoints can be adequately defined and determined). Whether the correlation will be exact remains to be seen. But it is clear that in those research or clinical areas where flicker-fusion shows interesting and/or useful results, QSD may make a contribution. For example, visA-waves might be helpful in those patients in whom the subjective CFF measure is unreliable, such as Parkinson's [4-6]. Even where the patient's CFF is reliable, the objective measure of visA-waves by QSD might augment or replace the psychophysical measurement of CFF in a variety of clinical conditions, such as migraine, Alzheimer's, reading disabilities, hypertension, drug side-effects, and visual deficits [7-11].

### Latency shift?

It is of interest that there seems to be a latency "shift" in the peaks of the larger A-waves when comparing waveforms at rates above and below the STZ (Figs. 4, 6, 8, 9, 10, 11). Such a shift can only be *known ****for certain ****by research which shows which peaks in the subSTZ and supraSTZ waveforms are functionally comparable*. But, ***for the purpose of this section***, *let's assume *that such *latency differences are present*. Given that we have associated subSTZ and supraSTZ waveforms with different *memories *(fusion-memory and flash-memory), it is but a small additional leap to consider whether the differences *which are a function of repetition-rate *are somehow connected with some mechanism that we will ***imagine ***as being *similar to *the spike-timing dependent plasticity of LTP (Long-Term Potentiation) and LTD (Long-Term Depression).

At excitatory cortical synapses, induction of synaptic plasticity is dependent both on the rate and the timing of input activities. While experimental protocols for study of these phenomena tend to emphasize the timing of activation rather than the rate, it is clear that both are jointly responsible for the induction of synaptic plasticity [25,26]. While this plasticity is generally studied and conceptualized with respect to changes lasting minutes to hours, in *our model *we assume that the *mechanisms *that trigger these longer effects *may also trigger shorter memory mechanisms, as well*. So, we note that, with respect to rate, LTP occurs with higher stimulation rates (e.g., 60–100 S/s), while LTD occurs with low rate stimulation (e.g., 13–20 S/s). The sign of plasticity (LTP or LTD) is dependent on the temporal order of synaptic activity relative to the back-propagation of the action potential. This temporal order might be affected by the latency-shifts we are assuming. The magnitude of the shift that we "eyeball" between the subSTZ and supraSTZ waveforms is 70–80 ms. This is of an appropriate size to move from the LTD window (75–50 ms before the action potential) to the LTP window (10–15 ms after the action potential) [25,26]. Hence, it is conceivable that cellular mechanisms could be triggered by the latency shift that distinguishes subSTZ and supraSTZ responses, and by implication might distinguish fusion and flash memories.

Since we are far out on a speculative limb, the incremental risk of further speculations seems small:

1) The effects triggered by the LTP/LTD mechanism with respect to fusionmemories would be predicted to be very short (if not enhanced by attention, emotions, etc.), such as less than 75 ms.

2) We wonder whether the time needed from the start of a rapid stimulus train, to develop the supraSTZ waveform (Fig. 15) should have some equivalent time at the cellular level. Such equivalent time might be the time necessary for activity in the dendritic tree, at higher input frequencies, to induce a prolonged depolarization in the cell that, together with continued synaptic activity, induces LTP [26].

3) Since the senior author hypothesizes that evoked-responses obtained at stimulus repetition-rates above about 6 S/s are almost entirely due to action potentials, he cannot resist commenting here that an increased peak latency of A-waves at supraSTZ firing rates (as controlled by stimulus repetition-rate) could be a measure of timings of the action potentials causing the back-propagation required for the "LTP-triggered" model suggested here. If so, then some aspects of the timing of these cellular processes could be detected and measured on the human scalp for research and clinical purposes. Note, however, the large number of "if's" needed to reach this notion.

4) The senior author also conjectures that the EEG may be the "ground" brain activity which registers the "current status" of unchanging sensory inputs via A-wave oscillations time-locked to the steady firing-rate of an given sensory input (as a function of the steady stimulus intensity at each sensory input, independently). When a changing sensory input results in a markedly-uneven firing rate, then the "figure" thus identified is rapidly analyzed with the brief A-wave responses (below STZ). The analysis involves associative memory.

Unclear to the senior author at this stage of the investigation are the following:

a) Does the large amount of "ground" activity affect the affect the associative memory search?

b) Is "ground" the "context" of the resulting association?

### The "oscillatory nature" of A-waves

A-waves indicate a new source of data about brain activity, obtained by a technique that can directly stimulate and record sustained oscillations of more than 1000 ms after each stimulus in a rapid stimulus train. This data may contribute to global theories of brain function that utilize "oscillations" as a generalization underlying many aspects of brain functioning, as described in several books [27-31]. Some articles report research on brain oscillatory behavior based upon EEG or ERP data, *e.g*., [32-34] while other articles describe theoretical approaches, *e.g. *[35-37]. Our data suggest that QSD methods may be applied across a considerable range of studies directed toward understanding neural oscillations, with the hope that this new approach may complement and deepen the interpretation of previous results and hopefully uncover new phenomena.

### A-waves as probability functions

At first thought, an "oscillation" might seem to indicate repetitive firing from neurons driven by the stimuli. Indeed, we described such neurons with reference to Fig. 21-Right. There is much evidence to indicate that PNS cells can be driven in timing with the repetitive stimuli, as can CNS cells that are innervated by such cells. But as one ascends the neurons of a sensory system, towards the cortex, it becomes more and more difficult to achieve a simple one-to-one correspondence between the timing of a simple stimulus and the timing of the cellular response. Such observations are relevant to considerations of what cellular activity underlies scalp-recorded A-waves.

**Figure 24:**
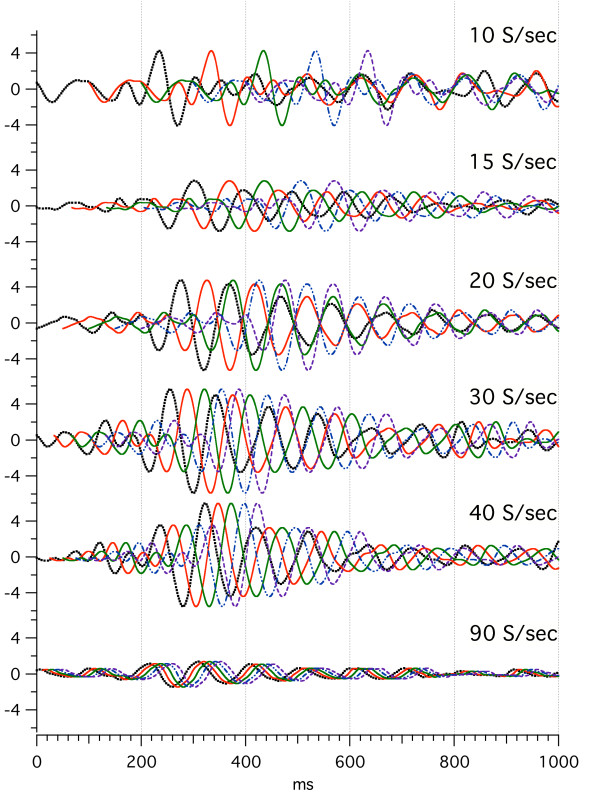
Simulation of overlap of visA-waves at different repetition-rates. The black dotted lines are the same data as shown in Fig. 4. Each waveform is duplicated and moved to the right by a distance equal to the mean repetition-rate for that waveform. This is repeated 4 times, so that the overlap of 5 successive responses are shown. *Note: *that there are multiple places where the peak from one stimulus overlaps a different peak from a different stimulus. These could be locations at which a given neuron could not fire at the same phase of *every *cycle. Note further that this is a simulation because there is not SI jitter, and that only 5 of the responses are shown, whereas in the experiments the stimuli were continuously presented.

If we assume that a given cortical cell fires at the same phase of each cycle in a sustained A-wave oscillation, there might be some stimulation rate at which the cell is about to fire due to the most recent stimulus, but has just fired as a later "cycle" to an earlier stimulus. In such a case, the refractory period of the cell may prevent a response to the most recent stimulus. A simulation of such a possibility is shown in Fig. 24, where it can be seen that there are multiple opportunities for this "conflict" to occur. However, we might not be able to detect a loss of such a response because our data is formed from averages of hundreds of stimuli, and responses from many thousands of cells. (We have proven [14], that variation in *signal ***cannot be detected **in the poor signal-to-noise conditions under which we record A-waves.) From such considerations, it may be a better "mental model" to imagine A-waves as representing the probability of synchronous firing in populations of cortical cells.

If such a model is accepted, what is the significance of a negative potential, in contrast to a positive potential, as a measure of probability? The senior author has previously shown that an AP (Action Potential) will produce one polarity at far-field electrodes when the AP is initiated, and the opposite polarity at the termination of the axon [38-40]. Since these potentials are dipoles rather than quadrupoles, these events are more easily detected at distant electrodes than is conduction along the axon (which can be quadrupolar). The consequence would be that the half-cycle time of the A-waves would be the conduction time from the initial segment to the axonal termination, as measured in a population of neurons. If the AP from neuron "A" activates neuron "B" and neuron B's AP travels subsequently in the opposite direction to the AP from neuron A, the initiation of the AP in B will have the same polarity as the termination of the AP of B. In such a case, the repetitive oscillations seen in A-waves are consistent with cyclic activity between two brain areas, such as could arise from thalamo-cortical or cortical-cortial reciprocal connectivity.

### "Can the brain really do THAT?"

We have received this type of comment from reviewers, and we feel it important to describe the limitations that affect *any *waveshapes that are obtained by *averaging*. It should be clear that an average may not represent any particular individual datum. Consider that although the mean number of children per family may be 2.3, there is no family with that number of children. This fact does not negate the usefulness of the mean value, but does limit its interpretation to the *population of families *rather than to any one family. So, while it is easy to imagine that the mean evoked-response occurs with *every *stimulus, this may not be the case. As mentioned in the previous paragraph, in the case of an initially poor signal-to-noise ratio, it is not possible to detect signal variation from run-to-run variation (see Appendix of QSD paper [14]). So the interpretation of the "meaning" of a waveform in terms of the neuronal generators which created it during a run of repeated stimuli may be different for different evoked responses. Note that these statements refer to *averaging*, which is the first step in QSD. Deconvolution of the *average *is the next step, but does not change the basic problem that has already been generated by the average. Said in another way: *QSD shares with averaging of evoked-responses the same ambiguities with regard to whether the average-waveform occurs with each stimulus or not*.

### "How can a nonlinear brain response be detected by a purely linear mathematical scheme?"

**Figure 25:**
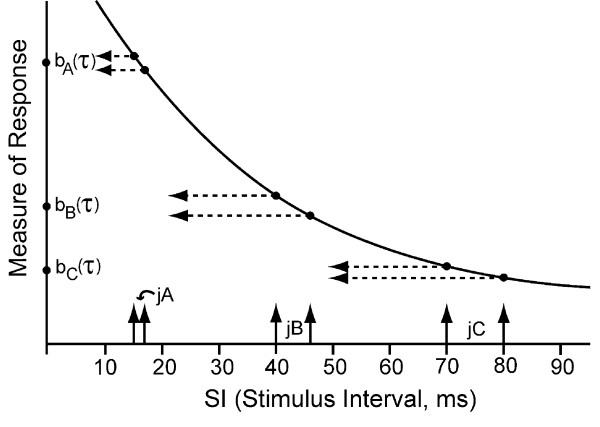
A nonlinear response is detected by repeated linear approximations by small excursions of the variable. (This figure taken from QSD methods paper [14].)

This is another reasonable question that we have received from reviewers. It is clear that A-waves are non-linear responses *with respect to stimulus repetition-rate*. It is also true that all computations in QSD are linear. However, as shown in Fig. 25, a nonlinear response can be detected by repeated runs in which the shape of the nonlinear response is estimated at a number of points, each using a linear approximation over a small excursion-range. This is a standard technique in physics and engineering. In our experiments, all stimulus parameters are kept constant during a run, except for the small excursion of the repetition-rate (12% jitter). The smaller the excursion, the more accurate is the estimate. The jitter excursions are somewhat smaller than the changes in repetition-rate necessary to show changes in A-waveforms.

### "Steady-State" Potentials compared with QSD waveforms

Starting in the early 90's, phenomena and theoretical excitement about the functional role of cortical oscillations (alluded to in the previous section), there was an expansion of the range of application of the SSVEP (Steady-State Visual Evoked Potential). The SSVEP was combined with cortical localization and topographic analysis, where the SSVEP was used as a "probe stimulus" that revealed activity in various areas of the brain under conditions of sensory and cognitive processing [41-44]. In the probe-SSVEP studies, the SS (Steady State) response amplitude is considered to vary inversely with intensity of processing in any area, according to the "processing capacity model" put forward by Papanicolaou [45]. The "spare" resources available to process the probe response go down as task processing load increases. This may have the same physiological mechanism as the well studied inverse variation of alpha amplitude with increased processing activity found in "Evoked Response Desynchronization" studies [46-52]. Several established researchers have developed the SS technique with their own technical variations and created new experimental designs [48,53,54] to apply the SSVEP to diverse fields of study [43,50-52,55,56], with clinical applications to areas such as migraine [57,58], schizophrenia [55,59], and Attention Deficit Hyperactivity Disorder [60,61].

Recently researchers have begun to compare the localization derived from electrical measures to localization using fMRI [48,62]. Techniques are now in use that permit simultaneous measurements of both SSVEP and fMRI [62]. Using these combinations of techniques [42-44,50,52,61,63-67], it is now possible to study:

1) Oscillatory neural processing over all parts of the cortex,

2) Cognitive processing from early sensory discrimination, recognition, and attentional processing, to complex cognitive tasks,

3) Working and long term memory as related to decision processes, and

4) Motor output sequencing and coordination. At the root of all this capability and these techniques is the use of the SS stimulation.

There are differences between the data presented in this paper and that obtained by SS stimulation:

1) The stimulus intervals in QSD are jittered, whereas in the SS response they are uniform.

2) The stimuli used in this paper, are brief, whereas "probe-SSVEP" stimulation uses sinusoidal stimulation.

Although these differences make direct comparisons between published results and ours problematic, the overlapped waveform average (i.e., the "raw data" before deconvolution) approximates to the SS average which would be obtained *using our brief stimuli *(with a uniform repetition-rate). For this reason, we call it the qSS (quasi-Steady-State) average. The peak *in the frequency-domain *at the stimulus repetition-rate in the qSS average has a peak that is equivalent to the SSVEP magnitude. So, if *experimental conditions *are similar, the results of the two methods can be reasonably compared in the frequency-domain.

**Figure 26:**
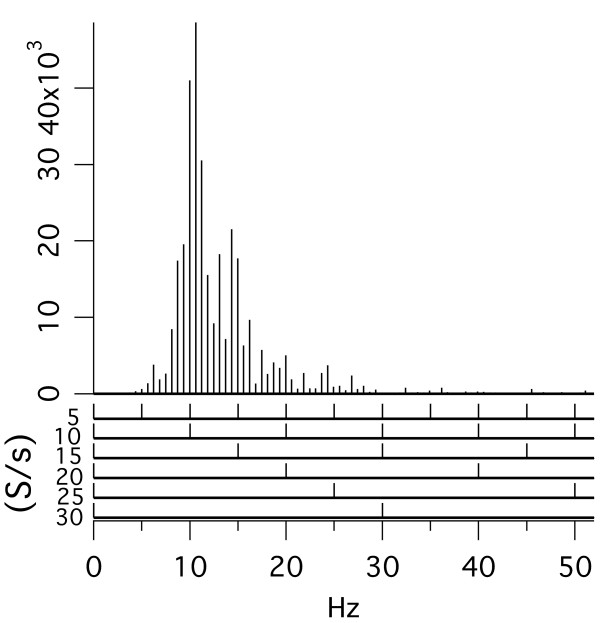
Frequency-domain plot of a visA-wave. The time-domain waveform is shown in Fig. 4, at 30 S/s. The 6 frequency-domain comb-filter amplitude plots at the bottom are those for uniform stimulus repetition-rates at the repetition-rates indicated.

Another method for comparing QSD visA-waves with SSVEP results is to simulate the SSVEP result-magnitude using *frequency-domain analysis *of the visA-waves, as we will now do. In Fig. 26 we show the frequency-domain power of the deconvolved time-domain visA-wave shown in Fig. 4 at 30 S/s. Note that the time-domain data used to compute Fig. 26 is circular, so that there is no distortion due to windowing; the frequencies are those of the signal, within the passband 8–50 Hz. In this frequency-analysis the prominent peak is just passed 10 Hz, with lesser peaks in the range of 13–17 Hz,*even though the stimulus repetition-rate was 30 S/s*.

**Figure 27:**
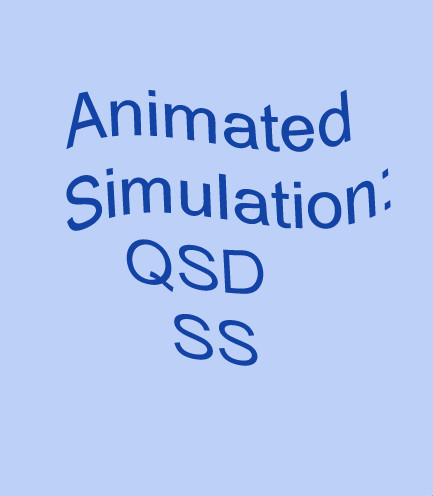
Animated simulations: SS compared with QSD. Herein you can access 6 simulations, 3 each for SS and for QSD. There are three ranges of mean repetition-rate: **A **= 0.3 – 2.4 S/s. **B **= 3 – 11 S/s. **C **= 11 – 25 S/s. Each of these rates can be seen for either SS or QSD from the following Demonstration files:"Fig. 27_SS_A" [see Additional file 13]"Fig. 27_SS_B" [see Additional file 14]"Fig. 27_SS_C" [see Additional file 15]"Fig. 27 QSD_A" [see Additional file 9]"Fig. 27 QSD_B" [see Additional file 10]"Fig. 27 QSD_C" [see Additional file 11] **SS Animations **These animations contain simulations of SS responses based upon an actual brain response waveform, also seen in Fig. 4, 30 S/s. That response is shown as a red trace in the lower left-hand side of all the steady-state animations. Above that is shown a 500 ms SS response in black, this is the same epoch length as used by Herrmann [16] and is equivalent to his averaged SS responses. The long blue trace shows the convolution of the brain response shown in the lower left, with a periodic sequence at the rate shown by the number in the top left. The first five seconds of our simulated convolved response are shown in the upper blue trace. In the bottom right hand corner there is a box that contains information plotted in the frequency domain. This box contains the frequencies from 0 Hz to 26 Hz with a mark below the horizontal axis showing 10 Hz. The red trace in the box is the magnitude of the Fourier coefficients of the time-domain brain response shown in the bottom left red trace. The blue dots are the Fourier coefficients of the blue trace above. The black vertical lines are the Fourier magnitudes of the periodic sequence (comb filter) with which the brain response is convolved. NOTE: All of the traces in these animations may have been scaled, and/or cropped for demonstrative purposes. **QSD Animations **These animations contain simulations of QSD responses based upon an actual brain response wave form recovered with the QSD method, also seen in Fig.4, 30 S/s. This response is shown in red at the bottom left. The long blue trace in the middle of the animation is the convolution of the brain response shown, with a QSD sequence at the mean repetition rate shown by the number in the top left. This trace is equivalent to our data-averages when stimulating with a QSD sequence. It is 5 sec long (longer than we have ever used) in order to show, in the simulation of the lowest stimulus repetition-rates the gradual overlap of the individual responses. In the bottom left, above the red trace is shown, in black, the corresponding waveform deconvolved from the upper blue trace (after random noise had been added). If we had not added noise here there would be no changes in the deconvolved trace during the animation. (Each of the three animations was based upon a different QSD sequence. QSD sequences for these simulations were produced by taking a QSD sequence used in this paper (see Table 1 [see Additional file 16]) and using it for multiple stimulus repetition-rates. To accomplish this author MO changed the sampling rate used during the simulation. (The frequencies are thinning as the repetition-rate goes faster *in the animation *because we did not want to find so many good q-sequences. So in the simulation, the use of one q-sequence over multiple frequencies led to automatic change in the length of the convolved data with every change in stimulus repetition-rate. This had the consequence that the number of frequencies analyzed changed, and this appears as changing q-sequence frequencies during the animation. In *actual practice*, since the SL is often the same length even though the repetition-rate is changed, the frequencies in the *deconvolution waveform****are the same***.) In the bottom right hand corner there is a box that contains information plotted in the frequency-domain. This box contains the frequencies from 0 Hz to 26 Hz with a mark below the horizontal axis showing 10 Hz. The red trace here is the magnitude of the Fourier coefficients of the brain's response shown in the red trace at the bottom left. The blue dots are the Fourier coefficients of the convolution shown as the blue trace above. The black vertical lines are the Fourier magnitudes of the q-sequence (i.e., Q-magnitudes) with which the brain's response is convolved (see QSD paper for further details) [14]. The tick marks on the vertical axis show the Q-magnitudes 1 and 5 of the q-sequence (cropped above 5). NOTE: All of the other traces in these animations may have been scaled, and/or cropped for demonstrative purposes.

We will now visualize the SSVEP results that would be obtained recording *this *brain response. As we have already proven [14], averaging overlapping waveforms is temporal convolution. In the frequency-domain, temporal convolution becomes just complex multiplication of the magnitude of the Fourier coefficients at each frequency in the frequency-spectra of the two circular vectors. So, if we want to know the *frequency-domain *result ***if ****the temporal waveform of *Fig. 4*(30 S/s) ****were uniformly convolved***, *we need only ****multiply ****the frequency-spectrum of the ****signal ***(Fig. 26) *by the frequency-spectrum of the uniform stimulus repetition-rate*, which is a ***comb filter***. The "comb filter" is so-named because the identical-height amplitudes in the frequency-spectrum of the uniform stimulus pattern look like the teeth of a gap-toothed comb. Comb filters for five uniform repetition-rate stimulation sequences, are shown at the bottom of Fig. 26.

Because the frequencies between the "teeth" of the comb filter are zero, the magnitude of the product resulting from the multiplication of zero times the visA-wave amplitude, no matter what it is, will be zero. Hence, there are no "results" from these frequencies, only from those frequencies that have "teeth". So we need only look at the products that will result at these frequencies. Starting with the comb for a uniform stimulus repetition-rate of 5 S/s, the first product will be very small, the second very large, the third about 50% of the second, the fourth *but a quarter of the third, and the rest being as small or smaller than the first*. These *products *are the totality of the frequency-domain information available from the time-domain average from the uniform repetition-rate. This limited information is too sparse to recover the time-domain waveform from the frequency-domain data.

Changing the repetition-rate merely changes the *"tooth frequencies" *whose limited number cannot reveal the details of the response. Nor can the magnitudes of different "tooth frequencies" observed by changing the repetition-rate be reasonably compared, *because they are probing different parts of the signal*. To better understand this, it is suggested that the reader repeat the process of identifying the parts of the signal-frequencies that are probed, for *each *of the stimulus repetition-rate comb filters shown at the bottom of Fig. 26. The reader can then confirm the following statements:

1) At 10 S/s, only two frequencies (10 and 20 Hz) contribute significantly to the products.

2) At 15 S/s and stimulus repetition-rates *above 15 S/s*, only the product at the stimulation frequency has much magnitude.

3) At a repetition-rate of 30 S/s, ***no frequencies of the response from 8–29 Hz ****(that were actually occurring when the brain was stimulated at 30 S/s) ****would contribute to the result! ***

4) If the usual practice in SS analysis were done, namely that *only *the product ***at the frequency of stimulation****is used*, then *the data obtained from the 6 runs at the bottom of *Fig. 26***would show marked variation in amplitude ****even though the ****actual ****brain response ****is the same in every run! ***

Thus, *if changes in amplitude of the "probe frequency"****occur ****as repetition-rate is changed*, one can conclude *either *that:

1) The response changed, or

2) The response didn't change (i.e., a different part of the response is being probed).

In consequence, the inherent information limits in SS data as a function of repetition-rate must be recognized. This error occurs when the experimental variable is **repetition-rate. **If the repetition-rate is held constant while some other variable is changed, then changes in the magnitude of the product at the stimulus repetition-rate *may *indicate changes in brain activity if the change in the experimental variable causes *no changes *in the general waveshape (time-domain), but only changes the magnitude of the entire brain response. But the waveshape must be determined using QSD, in order to validate such SS data.

(Technical note: The critique centered on Fig. 26 has not included the 1/N factor in Fourier Transformations, nor whether the magnitude of the comb filter varies with repetition-rate because of repeated use of the same sweep length in the average. The general conclusion would be the same, should these have been included.)

Because the limitations imposed by data collection *at a uniform rate *are important when considering SS data, we have animated the differences between SS analysis and QSD, as shown in demonstrations accessed from the Legend of Fig. 27. In each of these demonstrations, in the *lower left *is shown a red waveform which is the brain's response to the stimulus (time-domain). On the *lower right *(in the box) in red are the magnitudes of the brain's response in the *frequency-domain*. The vertical lines indicate the frequencies of the comb filter. Across the *top *is the *time-domain *data that will occur from repeated stimulation, as computed from the convolution of the comb filter and the brain's frequency-domain magnitudes. Recall that in an SS recording, this waveform *cannot be deconvolved*. On the other hand, in a QSD recording this waveform approximates to the SS recording, so we call it "qSS" (quasi-Steady State), and it *can be deconvolved*, as shown in the *middle left*. This waveform (middle left) is the time-domain waveform that occurs either as deconvolved brain response in QSD, or as a 500 ms window for SS.

As you watch the SS animations, you can see that as the repetition-rate changes, different frequencies of the brain response (lower right) make up the convolved waveform (across the top). As the stimulus repetition-rate gets faster and faster, the frequencies "probed" by the comb-filter become less and less, and the waveform of "the response" (middle left) becomes more and more simple, until it is just a sine wave (when only a single tooth of the comb-filter is within the frequency of the brain response).

In contrast, as you watch the QSD animations, you will see that the frequencies that are "probed" are *always numerous *because of the jittered sequence of the stimuli. Note that the deconvolved waveform recovered by QSD (middle left) is the same as the brain's response. In the absence of noise the two waveforms would be identical. Since you might not believe that we were actually computing the deconvolved waveform, we added some noise within the passband, so that the waveform changes slightly.

On repeated viewings, the reader can verify that whereas in the SS animations the convolved waveform becomes simpler and simpler as the repetition-rate increases, in the QSD animations there is continued complexity in the convolved waveform (across top). It is this complexity that the QSD method utilizes to recover the brain's response. The use of a uniform repetition-rate destroys such information.

### What we did not find

Like the dog that did not bark in one of the Sherlock Holmes' mysteries, what we did *not *observe may also be of some importance. Although oscillations with periods in the alpha-band were often observed,*** no ****prolonged or sustained oscillations in the**** gamma band**** were seen*. Note that in Fig. 3, G-waves show only 1.5 "cycles" in the gamma frequency range (between the peaks G0 and G2, and between the valleys G1.3 and G3). The G-waves are not prolonged oscillations, as seen in the A-waves. If the data is recorded with a passband of 30–120 Hz (as we have done for G-waves) then there can be summation of the 25 ms periods of the G-waves (peaks adding to peaks) since the larger A-waves are filtered out. If so, the decreased amplitude above and below 40 S/s *with this passband *can easily be due to peaks adding to valleys. Since we have recorded with an "open passband" in Fig. 19, one can see that the "G-wave portion" of the audA-wave recording is very small. So, if the "alpha-rate oscillations" seen in the open passband are removed by a high-pass filter, then the remaining waves may sum in the time-domain, as just described. If the observations of this explanation are replicated, then the lack of gamma activity in our recordings will be viewed in retrospect as not surprising. In which case we would have to conclude that 40 Hz may not be a critical stimulus repetition-rate *to whatever part of the CNS that is responding in synchrony to our jittered stimuli *. **Note however**, that this critique applies ***only to ****"40 Hz evoked responses" recorded from the scalp*, not to data from single cells or cell groups. Thus, we hypothesize that it is possible for *scalp-recorded *evoked-responses to *seem *to support single unit data, when the "support" is actually artifactual, based upon a fortuitous period between peaks in the ABR-AMLR, not upon cortical firings. **Note further, **that these comments do *not apply *to any *induced oscillations *which the stimulation may have caused and which we did not measure. ( What is notable is that some of our supraSTZ **stimulus repetition-rates **are in the gamma range. Thus, our results can be interpreted as showing long, synchronized "alpha waves" due to *prolonged stimulation**** at gamma rates*. **However, *the waveforms *obtained at these rates are *not unique *to "gamma-rate"*stimulation *since similar waveforms were recorded to "below-gamma" rates. Our only sure conclusion is that QSD methodology offers a new way to study stimulus repetition-rate effects in sensory systems.

## Conclusion

The data presented here is exploratory in nature, but the results, if confirmed in further research, could have important implications for both clinical electrophysiology and neuroscience.

For clinical electrophysiology, finding new CNS functionality that can be measured by scalp potentials opens new paths for detection of clinical abnormalities, even before the basis of the potentials is fully understood.

For neuroscience, the findings have implications which could change interpretations and require new experiments:

1) that stimulus repetition-rate can distinguish two different "modes" of CNS processing;

2) that these modes may differentiate ground from figure;

3) that these modes require two different memory mechanisms: fusion-memory and flash-memory;

4) that the character of these evoked-responses indicates a need for animal experiments in which both single-unit studies and evoked-response recordings are simultaneously recorded while switching "modes";

5) that these results show details that cannot be found with SS methods; and

6) that these findings provide a bridge between psychophysics and electrophysiology, in which the same phenomenon can be studied in the same subjects, at the same time.

Further information about this paper and topic is available online at [73].

## Methods

### Human Subjects

Adult subjects were recruited and gave informed consent in accordance with a protocol approved by our Institutional Review Board. One 17 yr old adolescent was also recorded after his parents gave their informed consent. None of the subjects had a history of epilepsy in themselves or family members. We often studied subjects who were being recorded under various other projects. Subjects normally came to the laboratory for more than one visit. Each visit could last for up to 5 hours. Short breaks and meals were scheduled in the session, and subjects were encouraged to request a break if fatigued. All data was coded with a two-letter identification that was unrelated to the subject's name, and these codings were used in this paper.

The subject's hearing was verified to be normal with a pure-tone audiometer, and vision by means of a Snellen chart. For visual studies we recorded from 6 subjects. We tried a large variety of stimulations in an exploratory mode, and took more than 100 runs, each requiring at least 10 min. From this set, the visA-waveforms in this paper were from 2 females and 1 male, age range 17–52 yrs. For auditory studies the data shown was selected from about 300 data runs, recorded in 21 subjects ranging in age from 21–73 years. The auditory runs usually took 40 min each. From this set, the audA-waveforms in this paper were from 5 males and 1 female, ages 17–26 yrs. For somatosensory recordings, one subject, age 74 years, was studied using electrical median nerve stimulation.

#### Methods, recording

The subjects sat semi-reclined in a chair with the head supported, to relax the neck muscles. The stimulus intensity was always comfortable, and subjects were asked to inform us if the stimuli seemed too bright or too loud.

Standard tin scalp electrodes were placed at C3'-O2 (where C3' is located halfway between C3 and Cz). Electrode paste was used for good contact after cleaning the skin with mildly abrasive gel on a Q-tip applicator. Potentials were amplified using battery-powered amplifiers from SA Instruments (Gain = 50,000) and then fed to the A-D converter (Swissonics) which connected to the computer via light-pipes. Recordings were acquired on a Mac G4 computer running MAX/MSP software, with A-D sampling at 48 kSamples/sec per channel, 24 bit accuracy with 100% duty cycle. The A-D was clock-coupled to the D-A (stimulus) (also 24 bit, at 48 kSamples/sec), and the D-A output also had 100% duty cycle. The data were stored direct-to-disk, for offline analysis. The usual recording time for visual stimulation was 10 min, during which 375 "sweeps" of a 1.6 sec timing sequence were placed on the computer disk. At a 10/sec stimulus repetition-rate, this is 6,000 stimuli, and at 90/sec it is 54,000 stimuli. The numbers would be 4 times larger for a 40 min auditory run. The usual filter settings of the amplifier were 1–500 Hz.

#### Methods, stimulation

For flash stimuli we used a Shimpo battery-powered digital stroboscope (model DT-315A) with an external trigger. The data-acquisition computer triggered the strobe with q-sequences. The strobe was mounted outside of one wall of the Faraday chamber, with the flash directed at a square aperture in the chamber. The subject listened to music via stereo headphones with source-electronics outside of the chamber, while fixating on a 1 cm diameter colored push-pin to the right or left of the square aperture, the aperture being 157 cm from the subject's eyes. When fixating on the pin the center of the white paper was 24° from the fovea. The aperture was covered with a blank piece of white paper to diminish the intensity. The dimensions of the white paper was 12 × 12 cm, which was 4.4° at the viewing distance (2.2° from center to edge). The mean luminance of the square was 1 cd/m2, with a range of 0.1 cd/m2 on repeated measurements.

Auditory stimuli were delivered by an Etymotic ER-2 tubephone, that used comfortable soft-sponge rubber ear-canal inserts. The intensity of the stimulation was adjusted according to the subject's hearing threshold and comfort level. Usually the stimuli were at an intensity of about 65 dB SL (threshold determined at slow rates). Stimuli were wither monaural 100 s clicks or increasing-frequency "Dau-chirps" [68], which covered a range of 500 Hz to 15 kHz and lasted about 6 ms. Zero time was set at the end of the chirp (when all the VIIIth nerve fibers are predicted to be in synchrony)*but did not include *the 1 ms delay in the ER2 tubing. Dau, et al [68] have shown that these chirps synchronize the VIIIth nerve firings better than other stimuli such as clicks or tone-pips.

Somatosensory stimulation was by electrical pulses 0.1 ms long, from a Grass S4 stimulator with stimulus isolation unit, at an intensity sufficient to cause the thenar muscles to contract. The stimulation was not painful. ( Stimulus sequences were previously determined as described in the QSD methods paper [14]. The sequences are given in Table 1 [see Additional file 16]. [The overall SL was chosen so as to cancel 60 Hz line interference when outputted at 48 kHz [14] In most of the cases the Q-magnitudes for a sequence were all above unity in the passband. In 5 cases the Q-magnitudes were below unity for one or two frequencies – in which case in the deconvolution the Q-magnitudes were "adjusted" to unity [14]. The sequences that were adjusted in this way were (see [see Additional file 16]): 12, 16, 35, 40 S/s (Fig. 8), and 20 S/s (Figs. 4 &6). This adjustment made no significant difference in the appearance of the time-domain waveform.

### Data analysis

Data was analyzed offline, first by averaging the raw data from the disk, and then by deconvolution calculations, as described in the Background, and in the QSD-methods paper [14]. Data analysis used a Mac G5 with our own software, which was incorporated into an IGOR (Wavemetrics) environment. Filtering was done after deconvolution, convolving the circular filter with the circular data. The filter was a Blackman-Harris window with the 3dB points placed at the stated passband limits. Thus, the q-sequence had Q-magnitudes greater than unity for the range of the passband, and usually for a few additional frequencies in each transition-band (at which the filter attenuation was the least). This filter minimized ringing in the time-domain.

## Note

There may be problems in reading some .mov files in this article when using the web browser safari. Such problems can be overcome by downloading to disk, or by using another browser.

## List of Abbreviations and Definitions

^© ^= the symbol used in this paper to denote the time-domain circular convolution.

*ABR *= Auditory Brainstem Response

*AEP *= Auditory Evoked Potential

*AMLR *= Auditory Middle Latency Response

*audA-wave *= "auditory-system A-wave"

*A-wave *= an evoked-response waveform with a latency starting at about 80–100 ms and whose duration is longer than the SI of the sustained stimulus repetition-rate used to obtain it. That is, the data is overlapped by the high stimulus rate.

*B(f) *= *b(t) *transformed to the frequency-domain.

*(f)*****= the estimated brain response, which contains noise, in the frequency-domain.

*b(t) *= the brain's evoked-response in the time-domain.

*(t)*****= the estimated brain response, which contains noise, in the time-domain.

*CFF *= Critical Fusion Frequency. The repetition-rate at which individual sensations"fuse" into a steady sensation. See also STZ.

*CNS *= Central Nervous System

*flash-memory *= memory that occurs when the SI is greater than about 150 ms, placing it as a subSTZ repetition-rate.

*fusion *= the psychophysical property when individual stimuli in a sequence cannot be distinguished. NOTE that for a given stimulus there can be a **range of fusion-boundary frequencies **because different aspects of the stimulus may fuse at *different *frequencies.

*fusion-memory *= memory that occurs when the SI is shorter than about 80 ms, placing it as a supraSTZ repetition-rate.

*G-waves *= evoked-responses with a latency of about 10–100 ms after the stimulus,*when obtained with a repetition-rate that overlaps the responses*.

*Hz *= Hertz. Cycles per second. In this paper it is used only in relationship to sine waves. (see S/s)

*jitter *= variation in SI in a q-sequence.

*LTP *= Long-Term Potentiation

*LTD *= Long-Term Depression

*N(f)*****= *n(t) *transformed to the frequency-domain.

*n(t) *= noise that contributes to the recorded signal, in the time-domain.

*PNS *= Peripheral Nervous System

*Q(f) = q(t) *transformed to the frequency-domain.

*q-sequence *= a Quasi-periodic timing sequence which has a small percentage jitter, and meets special frequency-domain constraints.

*QSD *= q-Sequence Deconvolution

*qSS *= quasi-steady-state. The potentials obtained by averaging when the stimulus repetition-rate is varied by only a small percentage by a q-sequence.

*q(t) *= the time-domain binary representation of the q-sequence, as a series of one's and zero's.

*SI *= Stimulus Interval (**start-**to-start) between successive stimuli. The SI is the time interval between two successive stimuli in a q-sequence. [To be distinguished from ISI (not used in this paper) which is the InterStimulus Interval (**end-**to-start.]

*S/s *= Stimuli per second. A measure of stimulus repetition-rate. In this paper, this unit is used, not Hz (q.v.).

*SS *= "Steady-State". This implies a uniform stimulus repetition-rate, with zero jitter, as contrasted with qSS.

*SSVEP *= "Steady-State Visual Evoked Potential"

*STZ *= Sensation-Transition Zone. The range of stimulus repetition-rates in which the sensation of "individual stimuli" changes to a "continuity".

*subSTZ *= sub Sensation-Transition Zone, i.e., a stimulus repetition-rate that is *below*the Sensation-Transition Zone.

*supraSTZ *= supra Sensation-Transition Zone, i.e., a stimulus repetition-rate that is *above *the Sensation-Transition Zone.

*VEP *= Visual Evoked Potential

*visA-wave *= "visual-system A-wave"

*(f)*****= *v(t) *transformed to the frequency-domain.

*(t)*****= the recorded activity from the scalp, including both brain activity and noise, in the time-domain.

## Declaration of competing interests

The support for the development of the QSD method came entirely from the National Institutes of Health. Most of the grants were under the SBIR program which requires commercialization. Abratech has patents on QSD. Researchers are invited to use QSD for scientific and other non-commercial purposes under a royalty-free license which can be obtained by registering at Abratech's website [73]. All other rights reserved.

## Authors' contributions

DLJ devised the QSD method, proposed looking for responses with the highpass filter below 30 Hz, analyzed the data, devised the various hypotheses, and drafted (and re-drafted) the manuscript.

TH independently devised and implemented the data acquisition and analysis software package that increased laboratory productivity; also analyzed the data and contributed to conceptualizing its implications, including a first version of the "dots" movie.

LLP contributed to the experimental design and data analysis, and brought out the connections to LTP/LTD.

BB participated in spirited debates over the implications of the data, and provided the correlations to the literature regarding oscillations and Steady-State responses.

MO aided in development of the filtering method and in solving other technical problems, independently found the correlations between visA-waves and visual after-potentials.

MT was crucial in inventing, producing, and perfecting the filtering method that is critically needed for QSD research within the alpha passband.

KM assisted with data analysis and interpretation, making sure that generalizations and claims about the data, so readily generated by the senior author, were accurate; also, she developed the "dots" movies out of a large collection of other "demonstrations" that proved ineffective.

PB provided important technical solutions for all parts of the data acquisition and analysis, and created the sound file demos.

All authors reviewed multiple drafts and provided comments for revisions.

## Supplementary Material

Additional file 1 "2 S/s (jitter)". Auditory stimuli (Dau-chirps) at 2 S/s with a 12% jitter. Click here for file

Additional file 2 "2 S/s (uniform)". Auditory stimuli (Dau-chirps) at 2 S/s at a uniform rate. Click here for file

Additional file 3 "100 S/s (jitter)". Auditory stimuli (Dau-chirps) at 100 S/s with a 12% jitter. Click here for file

Additional file 4 "100 S/s (uniform)". Auditory stimuli (Dau-chirps) at 100 S/s at a uniform rate. Click here for file

Additional file 5 MovieC. This is a QuickTime presentation of the dots of Fig. 20, at a high rate. Click here for file

Additional file 6 CONVOLVED DATA of Fig. 4. This is a collection of the convolved, averaged data from which the deconvolved waveforms of some of the figures were derived. Note that there is often a prominent 10 Hz appearance to these waveforms. The QSD-sequence must have Q-magnitudes greater than unity in the passband [14] This has the consequence that the convolution of the sequence with the brain's response waveform makes the 10 Hz response in *the convolved data****greater ***than in the response itself. This is corrected *in the deconvolution*, back to the correct magnitude for the brain's response [14]. Click here for file

Additional file 7 CONVOLVED DATA of Fig. 6. This is a collection of the convolved, averaged data from which the deconvolved waveforms of some of the figures were derived. Note that there is often a prominent 10 Hz appearance to these waveforms. The QSD-sequence must have Q-magnitudes greater than unity in the passband [14] This has the consequence that the convolution of the sequence with the brain's response waveform makes the 10 Hz response in *the convolved data****greater ***than in the response itself. This is changed *in the deconvolution*, to the correct magnitude for the brain's response [14]. Click here for file

Additional file 8 CONVOLVED DATA of Fig. 8. This is a collection of the convolved, averaged data from which the deconvolved waveforms of some of the figures were derived. Note that there is often a prominent 10 Hz appearance to these waveforms. The QSD-sequence must have Q-magnitudes greater than unity in the passband [14] This has the consequence that the convolution of the sequence with the brain's response waveform makes the 10 Hz response in *the convolved data****greater ***than in the response itself. This is changed *in the deconvolution *to the correct magnitude for the brain's response [14]. Click here for file

Additional file 9 QSD-A Click here for file

Additional file 10 QSD-B Click here for file

Additional file 11 QSD-C Click here for file

Additional file 12 MovieB. This is a QuickTime presentation of the dots of Fig. 20, at a slow rate. Click here for file

Additional file 13 SS-A Click here for file

Additional file 14 SS-B Click here for file

Additional file 15 SS-C Click here for file

Additional file 16 "Table 1" Table of q-sequences used to obtain the data in the Figures.
Note: If you wish to see an analysis of a q-sequence, you can click on the appropriate "Additional file" in *this *legend. The *name in this list *shows the repetition rate and the associated Figure numbers in which the sequence was used, in the following format: rate_Fig#. Each q-sequence data analysis will show:
**Upper left**: Each time between successive stimulus locations (called "plocs") in the sequence. (The "locs" in "plocs" refers to "locations". We can't remember what the "p" stands for.)
**Upper right**: A histogram of these times.
**Lower left**: The Q-magnitudes of the sequence in the passband (passband limits [marked by two vertical red lines]). All Q-magnitudes within these bounds are above the unity line (horizontal red line). Also shown in the passband is the mean Q-magnitude (blue line) and the root-mean-square (black line).
**Lower right**: A histogram of the Q-magnitudes in the passband.
**Lower right**: A histogram of the Q-magnitudes in the passband. 8persec_fig8 [see Additional file 61]
11persec_fig4_6 [see Additional file 62]
12persec_fig8 [see Additional file 63]
16persec_fig4_6_8_9_10_17 [see Additional file 64]
20persec_fig4_6 [see Additional file 65]
31persec_fig4_6_8_9_11 [see Additional file 66]
35persec_fig8 [see Additional file 67]
40persec_fig4_6 [see Additional file 68]
40persec_fig8 [see Additional file 69]
41persec_fig15_17 [see Additional file 70]
50persec_fig8 [see Additional file 71]
70persec_fig8 [see Additional file 72]
80persec_fig8 [see Additional file 73]
90persec_fig4_6 [see Additional file 74]
Click here for file

Additional file 17 2persec_click Click here for file

Additional file 18 2persec_dau Click here for file

Additional file 19 4persec_click Click here for file

Additional file 20 4persec_dau Click here for file

Additional file 21 6persec_click Click here for file

Additional file 22 6persec_dau Click here for file

Additional file 23 8persec_click Click here for file

Additional file 24 8persec_dau Click here for file

Additional file 25 10persec_click Click here for file

Additional file 26 10persec_dau Click here for file

Additional file 27 12persec_click Click here for file

Additional file 28 12persec_dau Click here for file

Additional file 29 14persec_click Click here for file

Additional file 30 14persec_dau Click here for file

Additional file 31 16persec_click Click here for file

Additional file 32 16persec_dau Click here for file

Additional file 33 18persec_click Click here for file

Additional file 34 18persec_dau Click here for file

Additional file 35 20persec_click Click here for file

Additional file 36 20persec_dau Click here for file

Additional file 37 22persec_click Click here for file

Additional file 38 22persec_dau Click here for file

Additional file 39 24persec_click Click here for file

Additional file 40 24persec_dau Click here for file

Additional file 41 26persec_click Click here for file

Additional file 42 26persec_dau Click here for file

Additional file 43 28persec_click Click here for file

Additional file 44 28persec_dau Click here for file

Additional file 45 30persec_click Click here for file

Additional file 46 30persec_dau Click here for file

Additional file 47 40persec_click Click here for file

Additional file 48 40persec_dau; "No jitter – uniform". Click here for file

Additional file 49 50persec_click Click here for file

Additional file 50 50persec_dau Click here for file

Additional file 51 70persec_click Click here for file

Additional file 52 70persec_dau Click here for file

Additional file 53 90persec_click Click here for file

Additional file 54 90persec_dau Click here for file

Additional file 55 100persec_click Click here for file

Additional file 56 100persec_dau Click here for file

Additional file 57 "MLS" Click here for file

Additional file 58 "12percent jitter" Click here for file

Additional file 59 "24percent jitter" Click here for file

Additional file 60 "36percent jitter" Click here for file

Additional file 61 q-sequence: 8persec_fig8 Click here for file

Additional file 62 q-sequence: 11persec_fig4_6 Click here for file

Additional file 63 q-sequence: 12persec_fig8 Click here for file

Additional file 64 q-sequence: 16persec_fig4_6_8_9_10_17 Click here for file

Additional file 65 q-sequence: 20persec_fig4_6 Click here for file

Additional file 66 q-sequence: 31persec_fig4_6_8_9_11 Click here for file

Additional file 67 q-sequence: 35persec_fig8 Click here for file

Additional file 68 q-sequence: 40persec_fig4_6 Click here for file

Additional file 69 q-sequence: 40persec_fig8 Click here for file

Additional file 70 q-sequence: 41persec_fig15_17 Click here for file

Additional file 71 q-sequence: 50persec_fig8 Click here for file

Additional file 72 q-sequence: 70persec_fig8 Click here for file

Additional file 73 q-sequence: 80persec_fig8 Click here for file

Additional file 74 q-sequence: 90persec_fig4_6 Click here for file
