# Supplementary figures and images for: Human sensory-evoked responses differ coincident with either "fusion-memory" or "flash-memory", as shown by stimulus repetition-rate effects
Source: BMC Neurosci. 2006 Feb 23;7:18. doi: 10.1186/1471-2202-7-18 (PMC1483834; doi:10.1186/1471-2202-7-18)

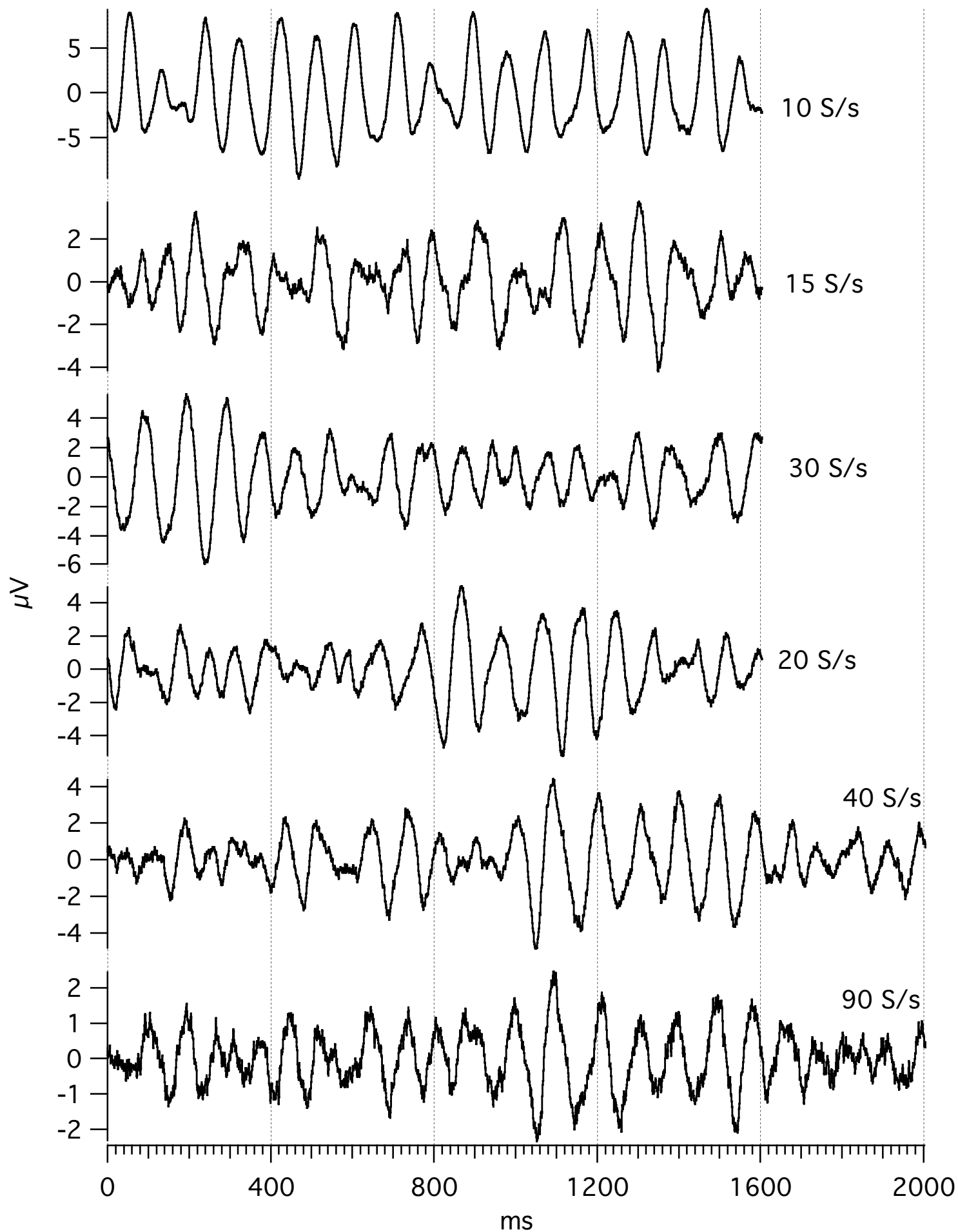

Supplement: Additional file 6 — CONVOLVED DATA of Fig. 4. This is a collection of the convolved, averaged data from which the deconvolved waveforms of some of the figures were derived. Note that there is often a prominent 10 Hz appearance to these waveforms. The QSD-sequence must have Q-magnitudes greater than unity in the passband [14] This has the consequence that the convolution of the sequence with the brain's response waveform makes the 10 Hz response in the convolved datagreater than in the response itself. This is corrected in the deconvolution, back to the correct magnitude for the brain's response [14]. [file 1471-2202-7-18-S6.pdf]

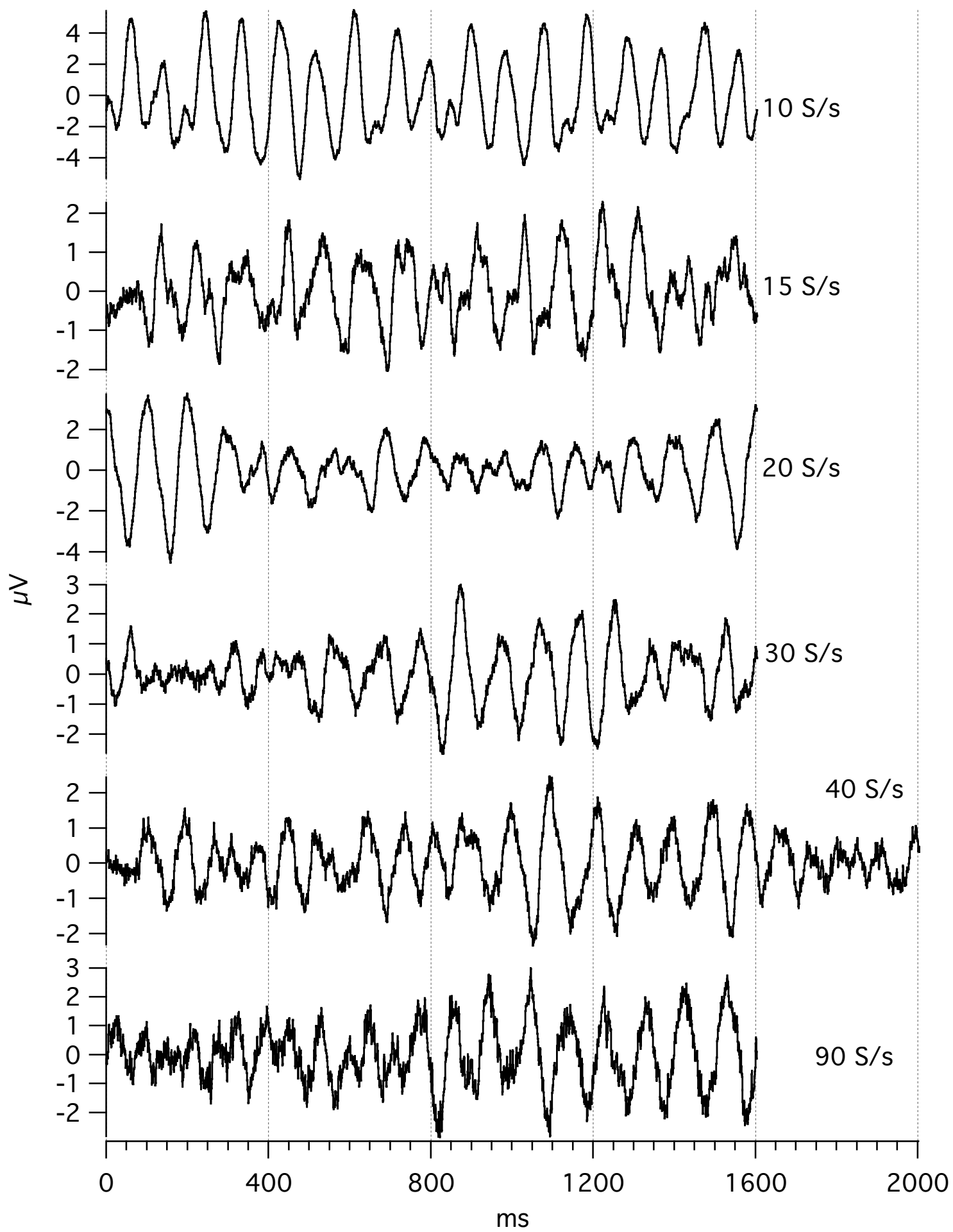

Supplement: Additional file 7 — CONVOLVED DATA of Fig. 6. This is a collection of the convolved, averaged data from which the deconvolved waveforms of some of the figures were derived. Note that there is often a prominent 10 Hz appearance to these waveforms. The QSD-sequence must have Q-magnitudes greater than unity in the passband [14] This has the consequence that the convolution of the sequence with the brain's response waveform makes the 10 Hz response in the convolved datagreater than in the response itself. This is changed in the deconvolution, to the correct magnitude for the brain's response [14]. [file 1471-2202-7-18-S7.pdf]

# Convolved Auditory responses

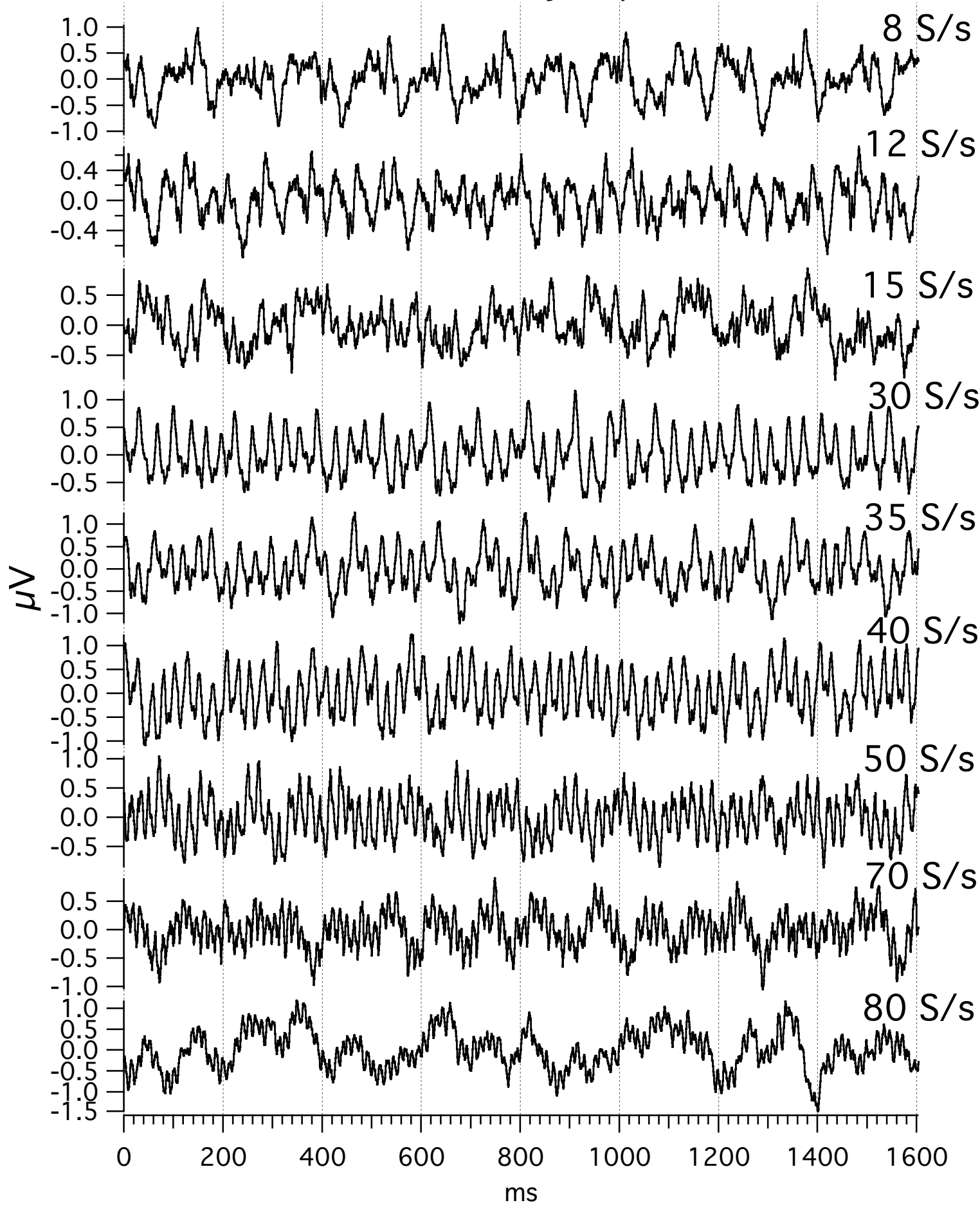

Supplement: Additional file 8 — CONVOLVED DATA of Fig. 8. This is a collection of the convolved, averaged data from which the deconvolved waveforms of some of the figures were derived. Note that there is often a prominent 10 Hz appearance to these waveforms. The QSD-sequence must have Q-magnitudes greater than unity in the passband [14] This has the consequence that the convolution of the sequence with the brain's response waveform makes the 10 Hz response in the convolved datagreater than in the response itself. This is changed in the deconvolution to the correct magnitude for the brain's response [14]. [file 1471-2202-7-18-S8.pdf]

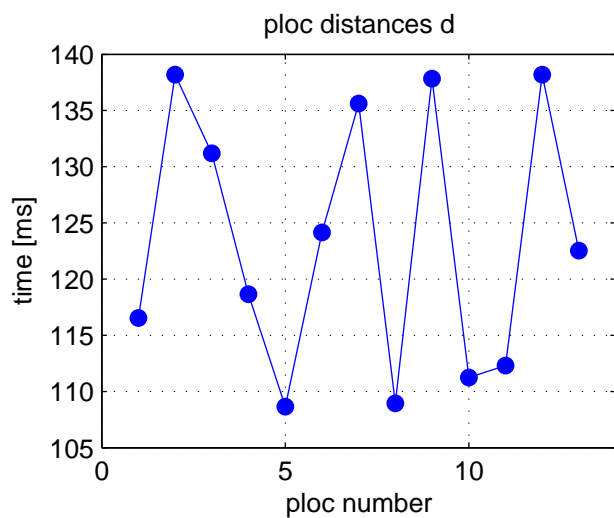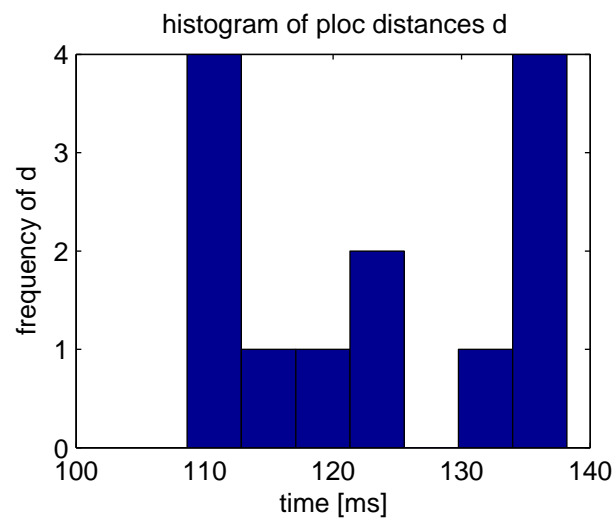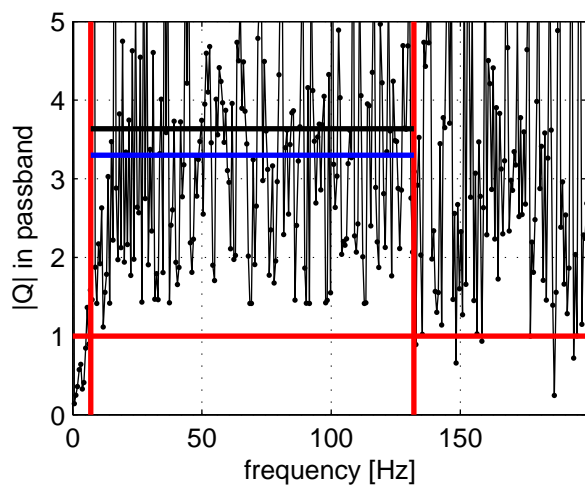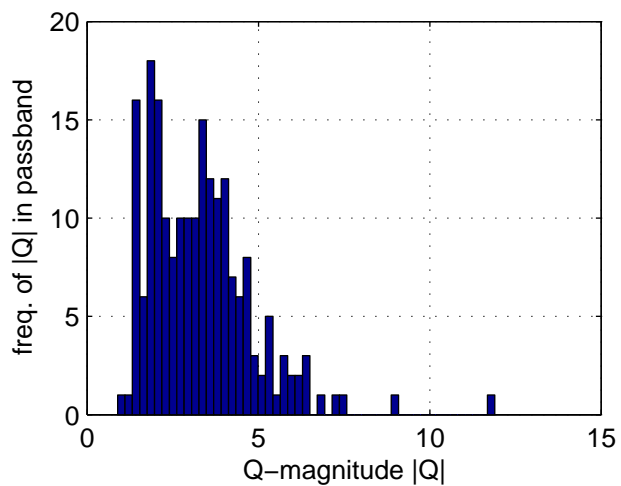

Supplement: Additional file 61 — q-sequence: 8persec_fig8 [file 1471-2202-7-18-S61.pdf]

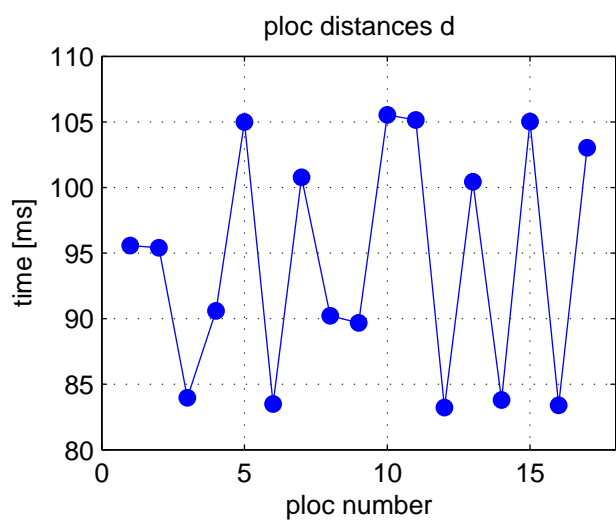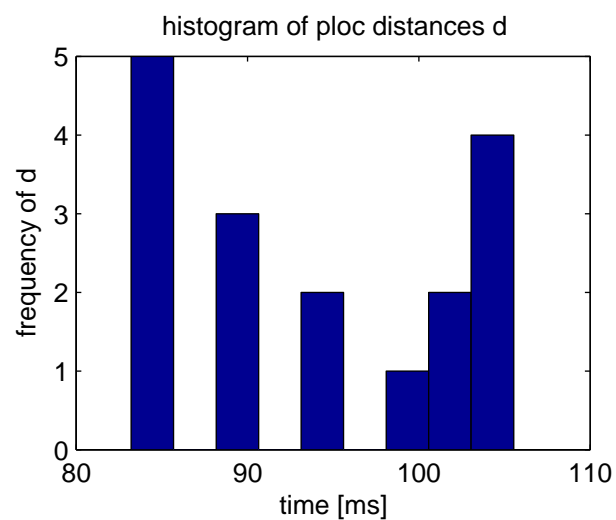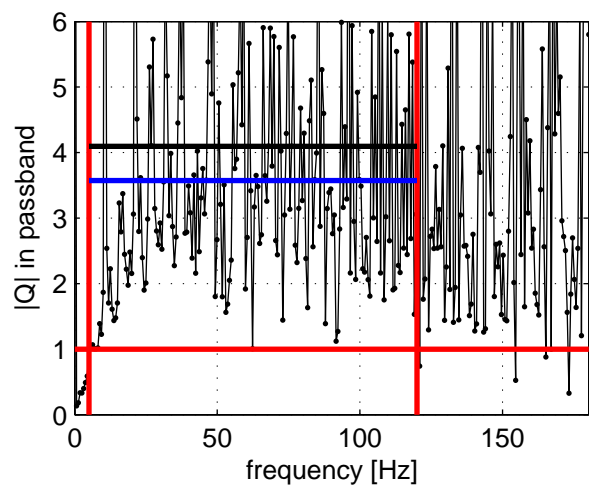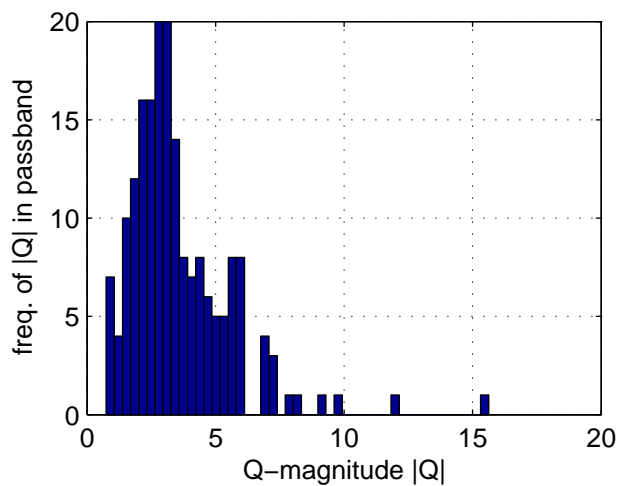

Supplement: Additional file 62 — q-sequence: 11persec_fig4_6 [file 1471-2202-7-18-S62.pdf]

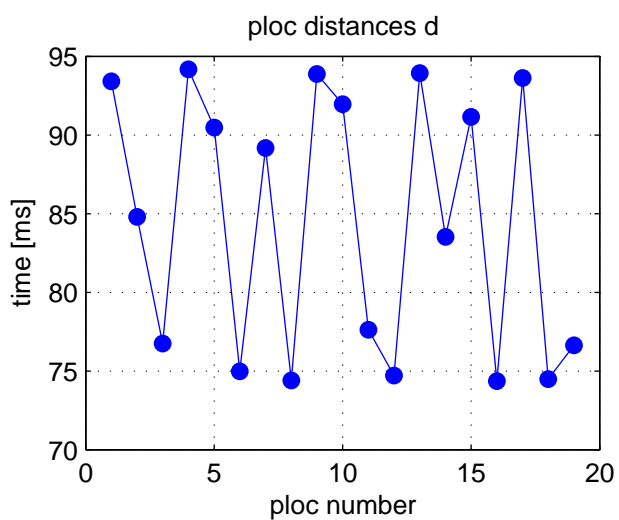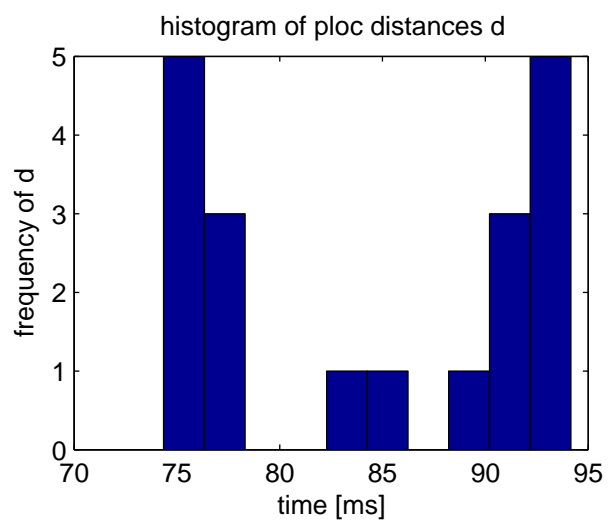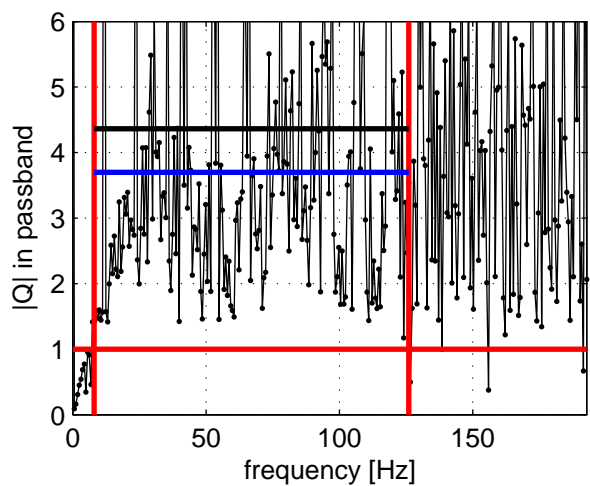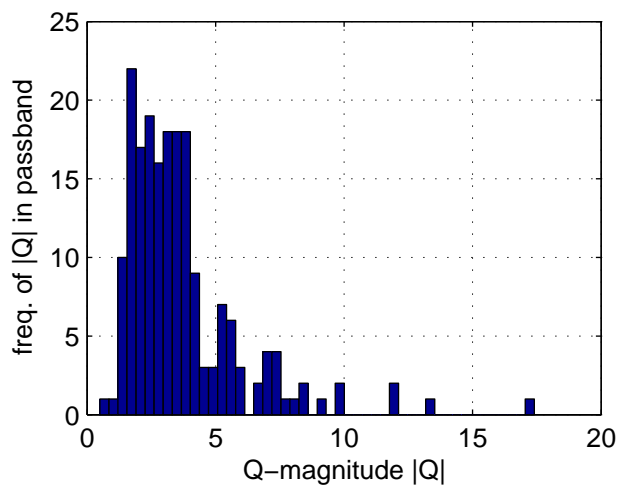

Supplement: Additional file 63 — q-sequence: 12persec_fig8 [file 1471-2202-7-18-S63.pdf]

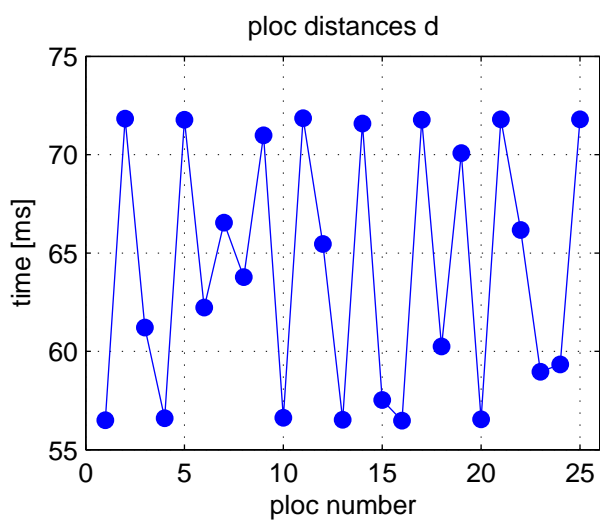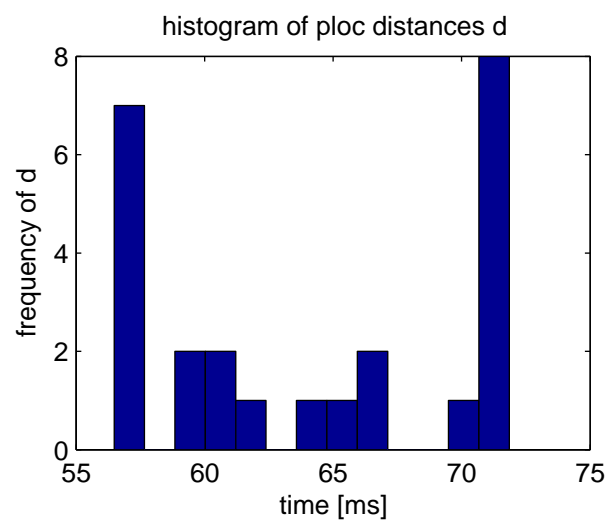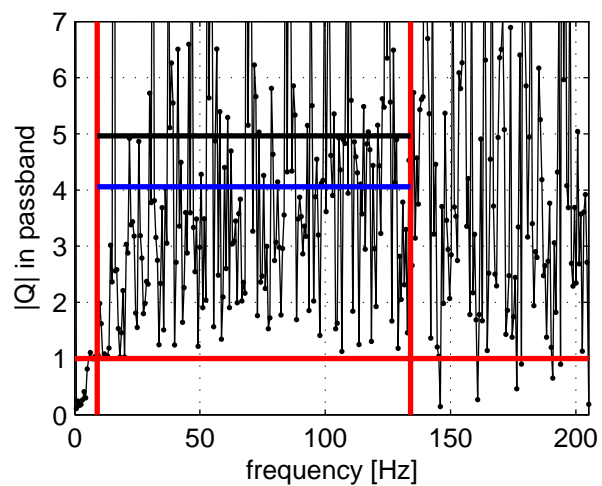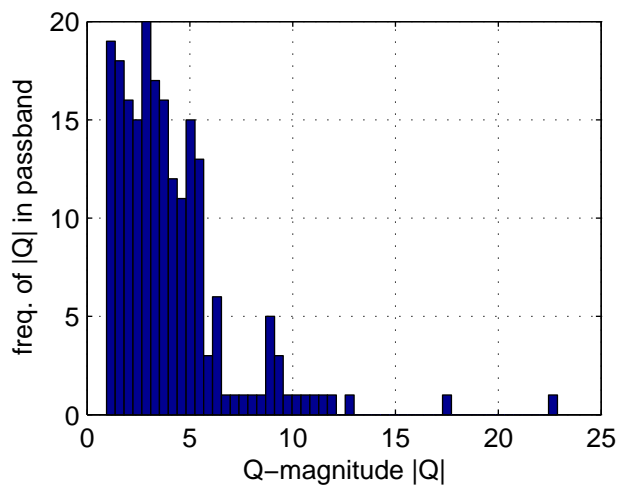

Supplement: Additional file 64 — q-sequence: 16persec_fig4_6_8_9_10_17 [file 1471-2202-7-18-S64.pdf]

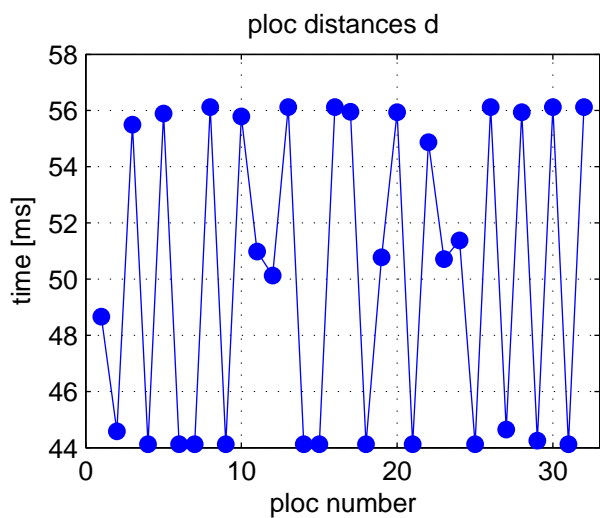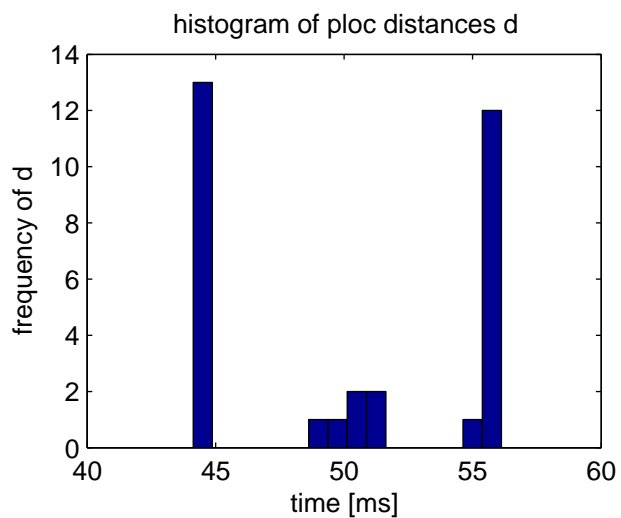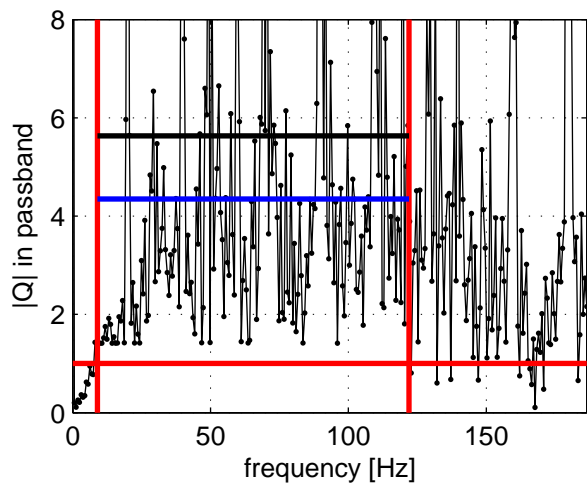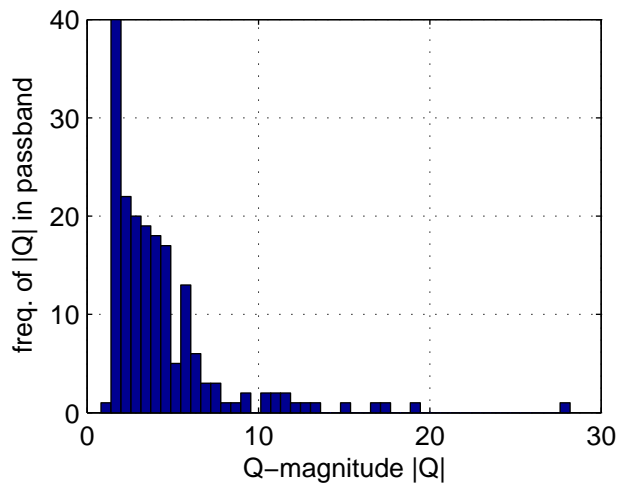

Supplement: Additional file 65 — q-sequence: 20persec_fig4_6 [file 1471-2202-7-18-S65.pdf]

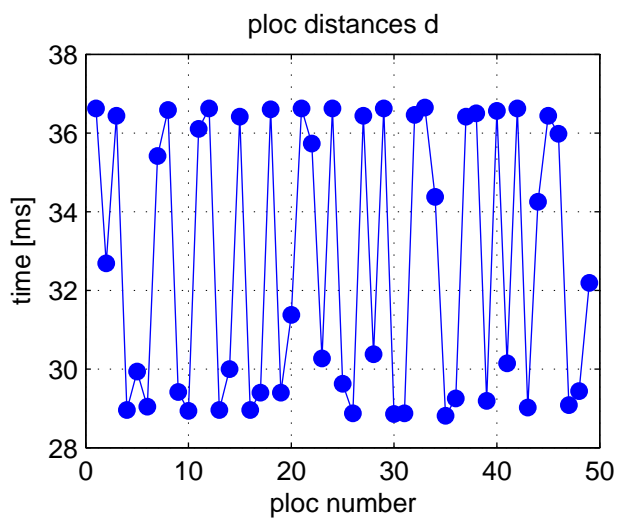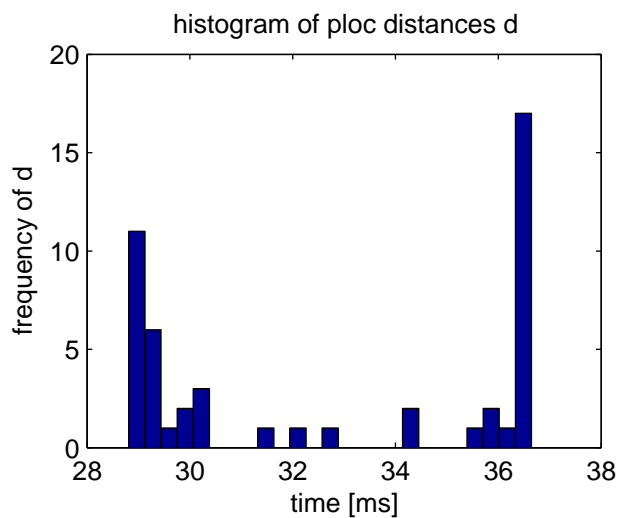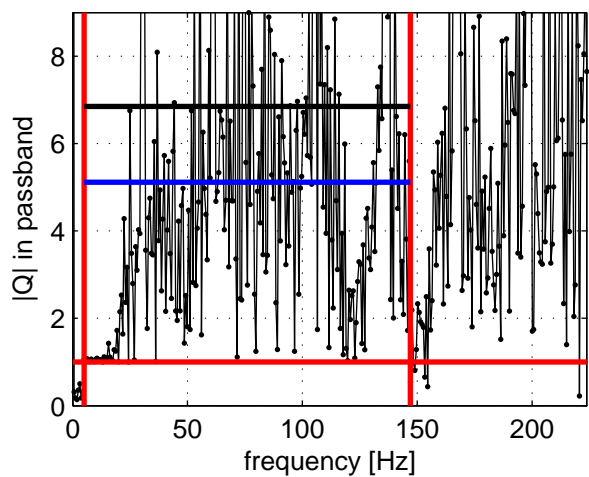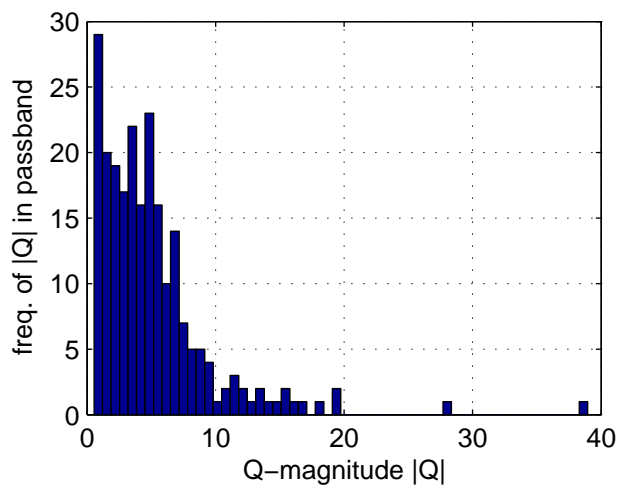

Supplement: Additional file 66 — q-sequence: 31persec_fig4_6_8_9_11 [file 1471-2202-7-18-S66.pdf]

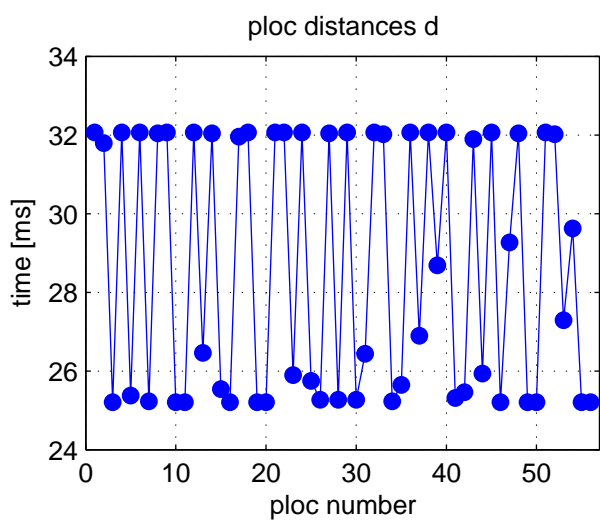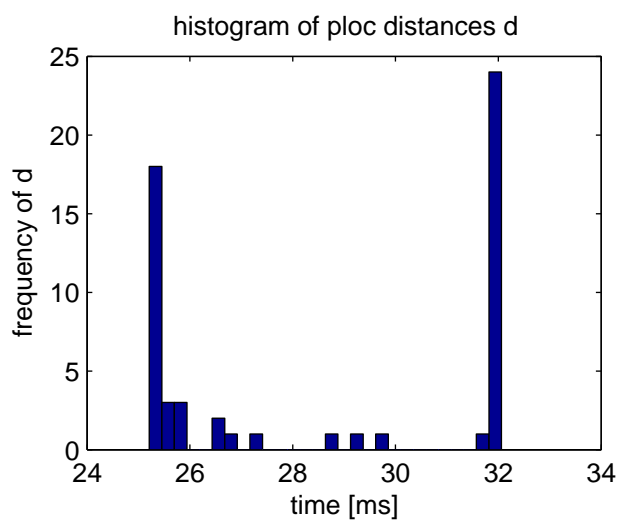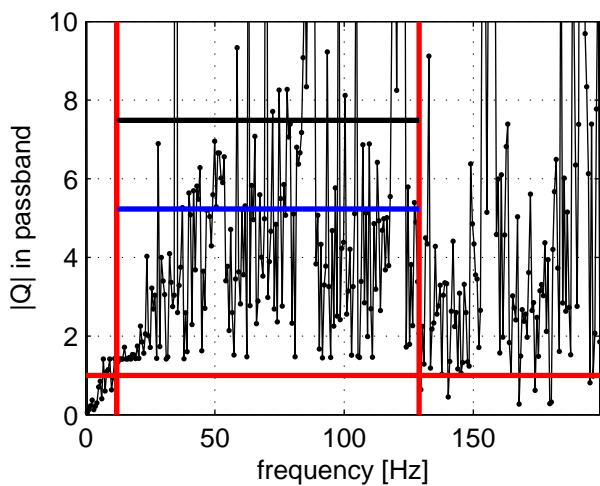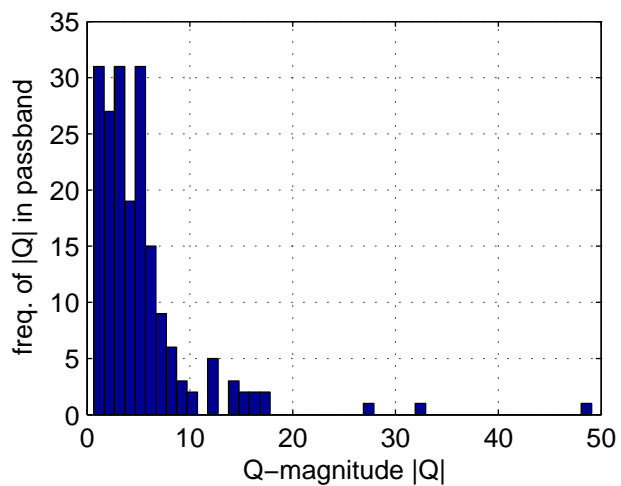

Supplement: Additional file 67 — q-sequence: 35persec_fig8 [file 1471-2202-7-18-S67.pdf]

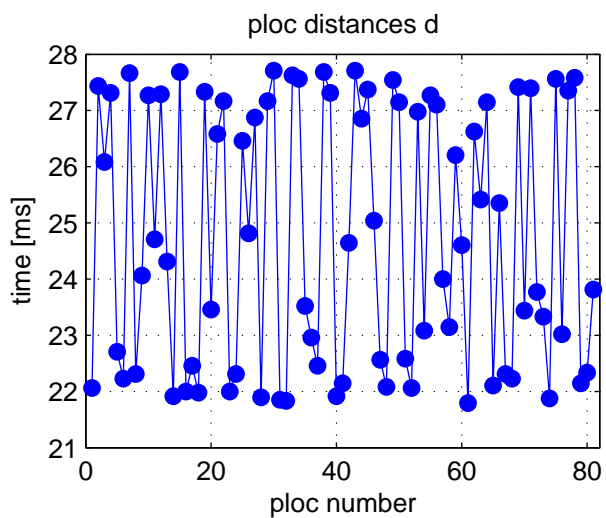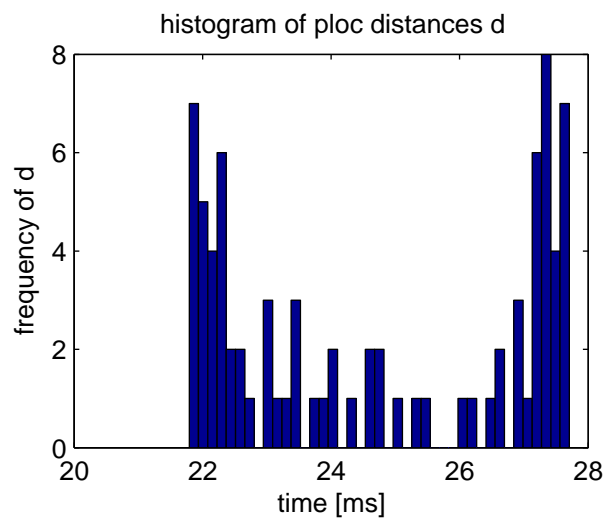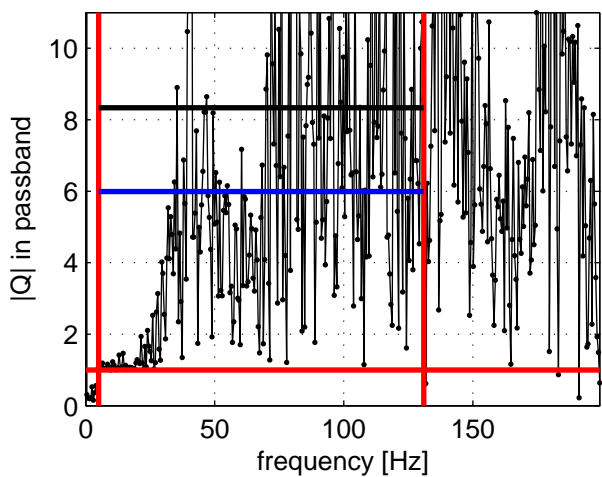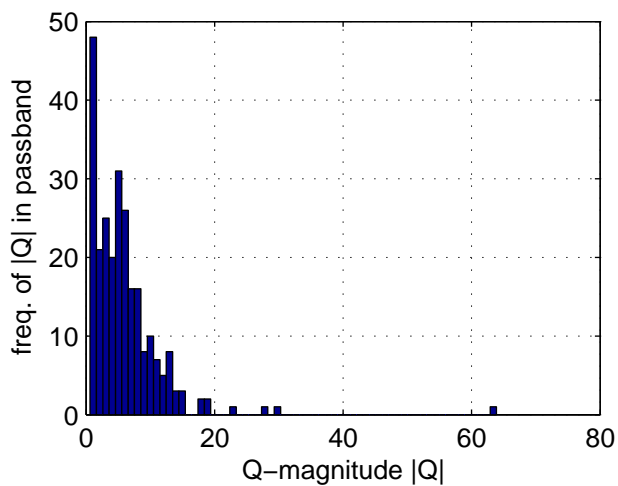

Supplement: Additional file 68 — q-sequence: 40persec_fig4_6 [file 1471-2202-7-18-S68.pdf]

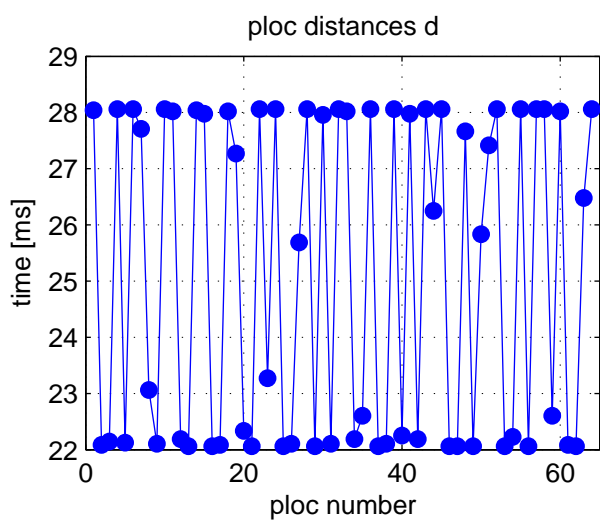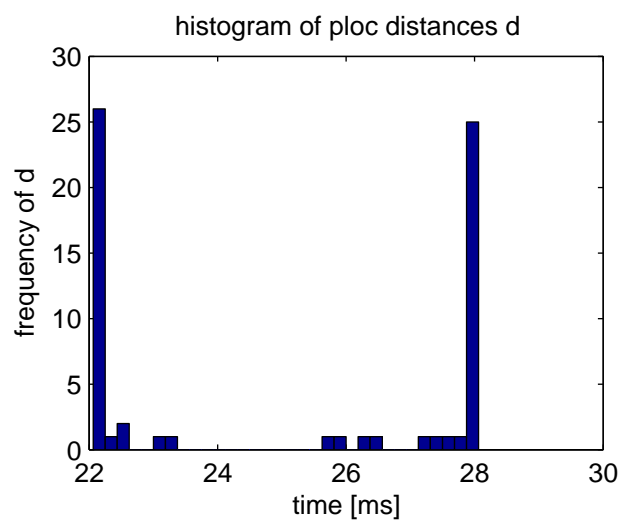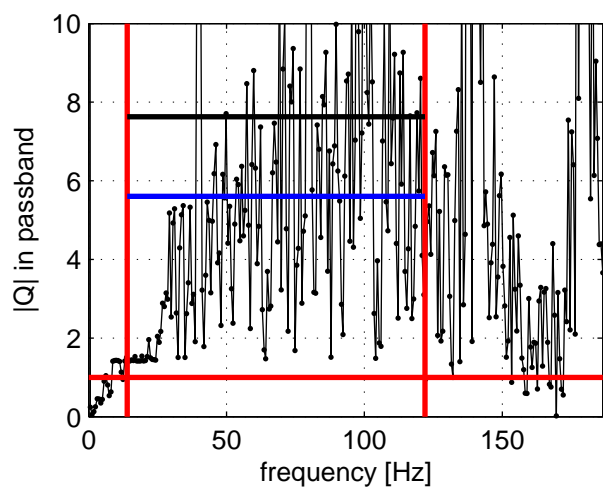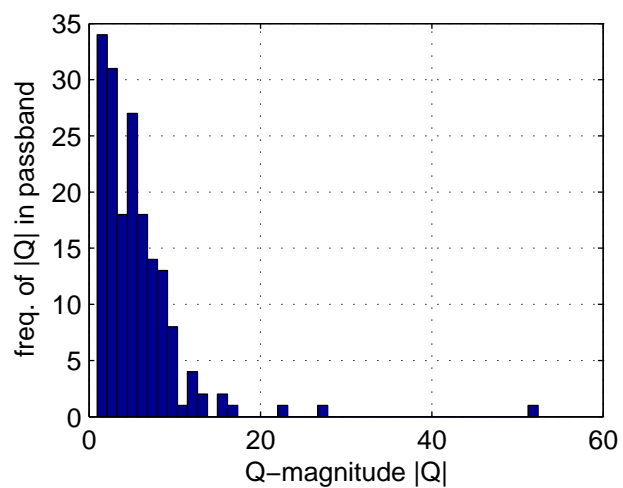

Supplement: Additional file 69 — q-sequence: 40persec_fig8 [file 1471-2202-7-18-S69.pdf]

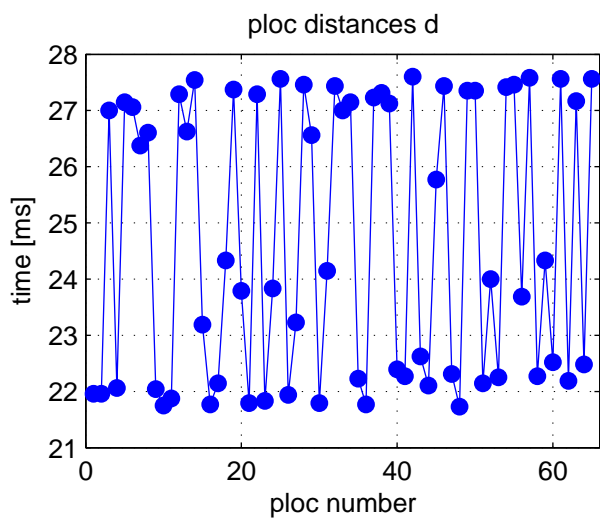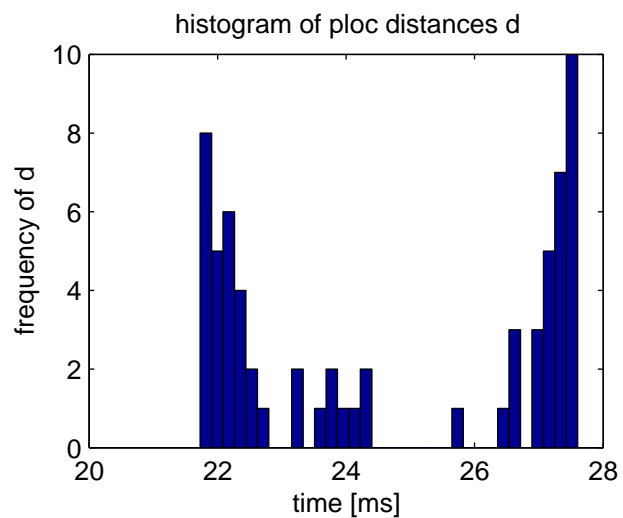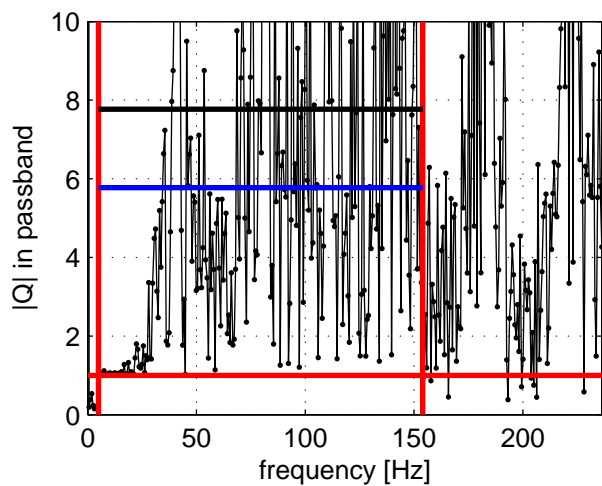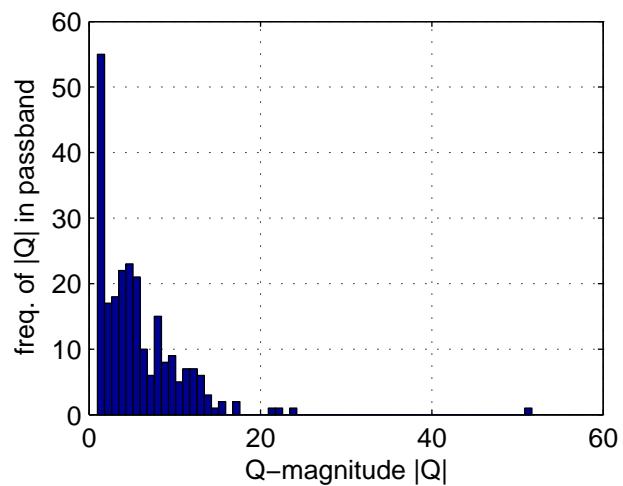

Supplement: Additional file 70 — q-sequence: 41persec_fig15_17 [file 1471-2202-7-18-S70.pdf]

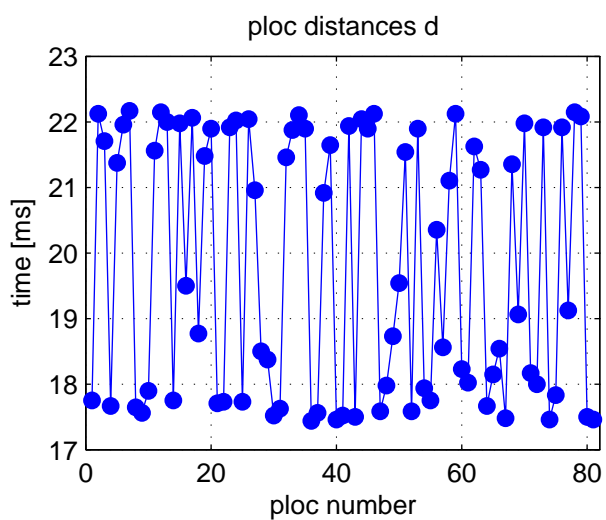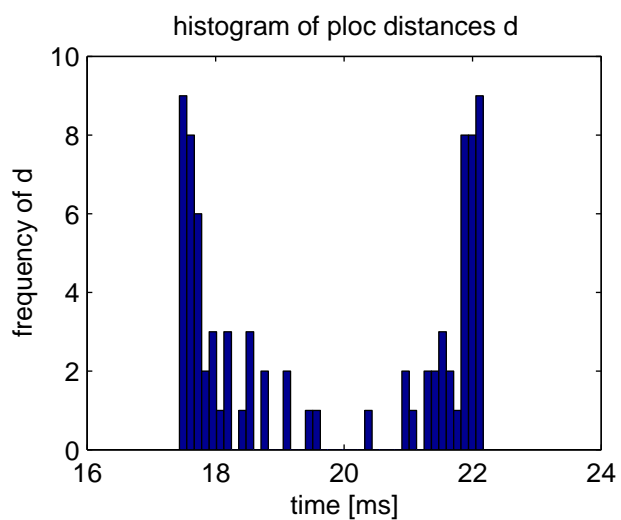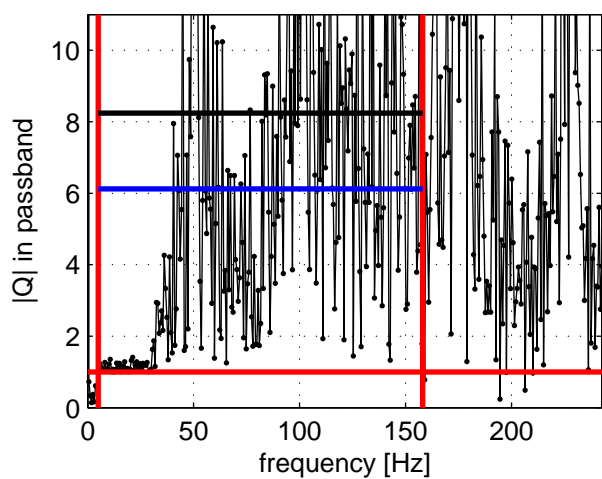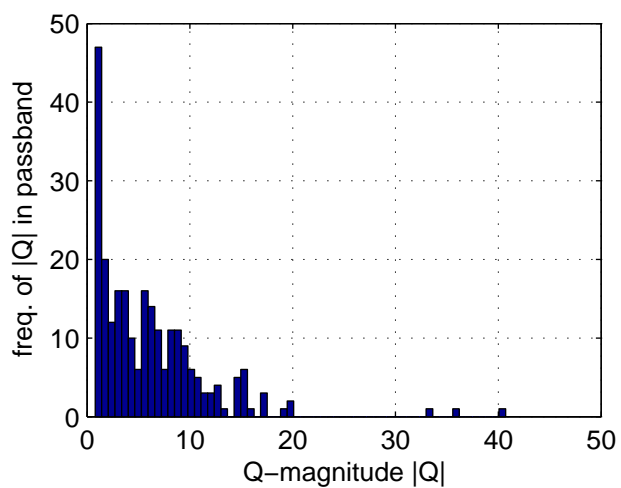

Supplement: Additional file 71 — q-sequence: 50persec_fig8 [file 1471-2202-7-18-S71.pdf]

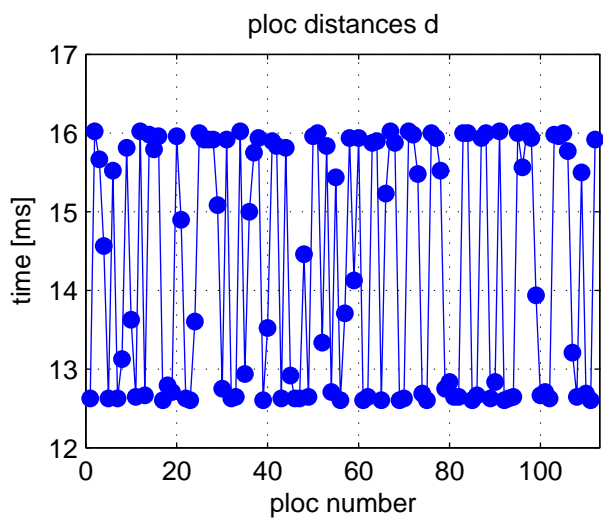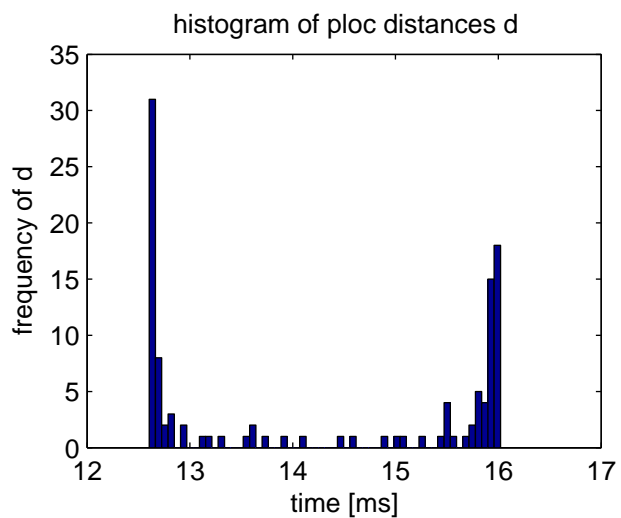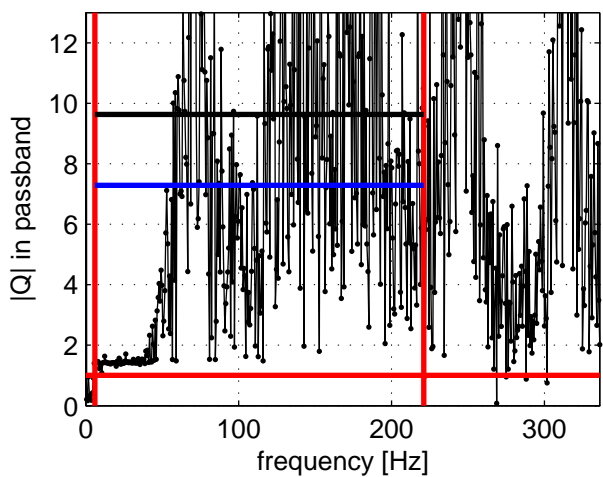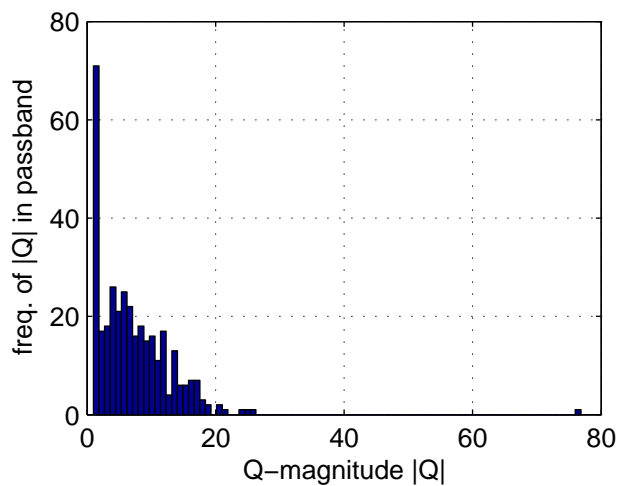

Supplement: Additional file 72 — q-sequence: 70persec_fig8 [file 1471-2202-7-18-S72.pdf]

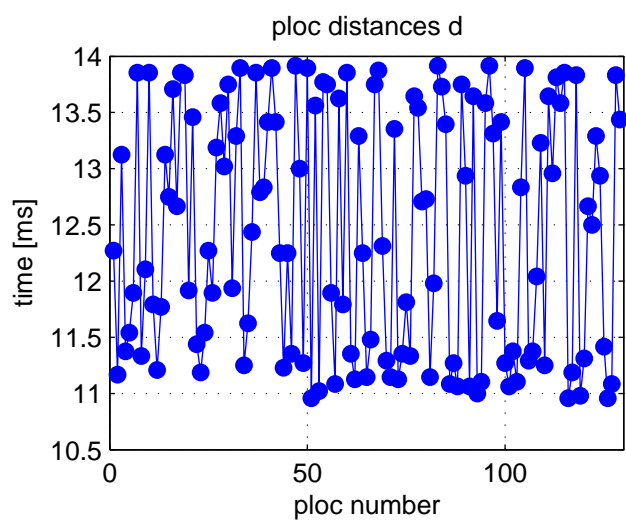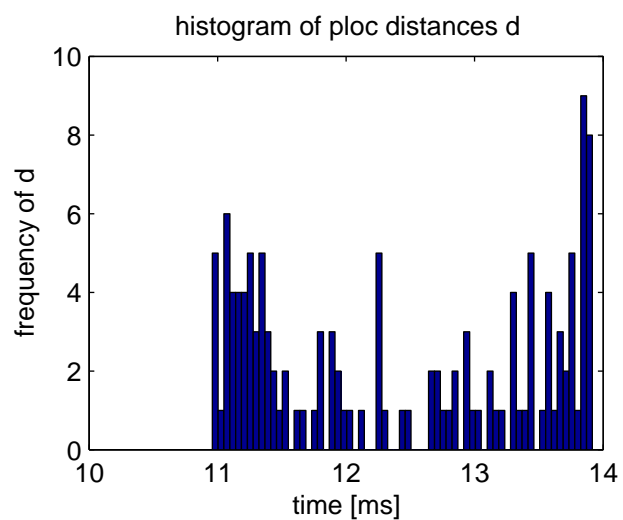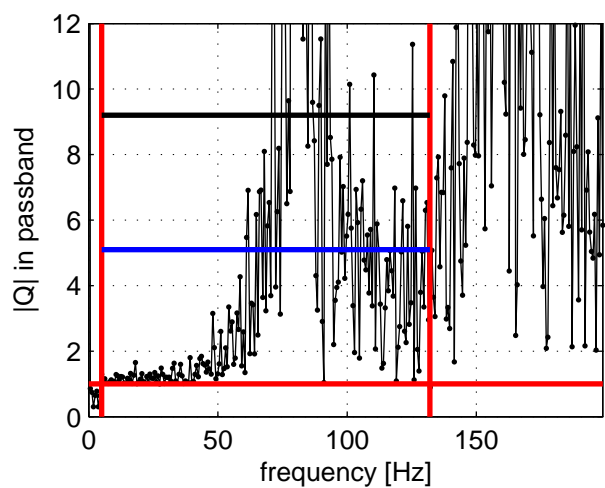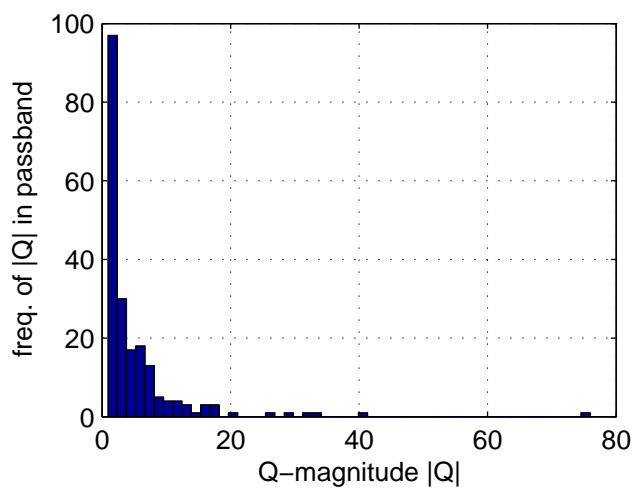

Supplement: Additional file 73 — q-sequence: 80persec_fig8 [file 1471-2202-7-18-S73.pdf]

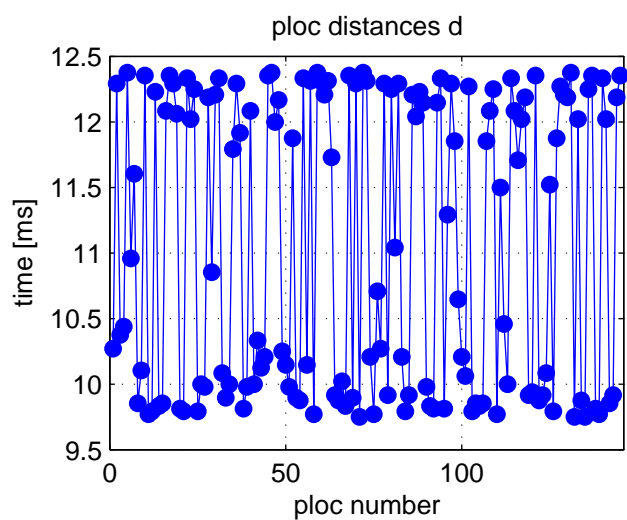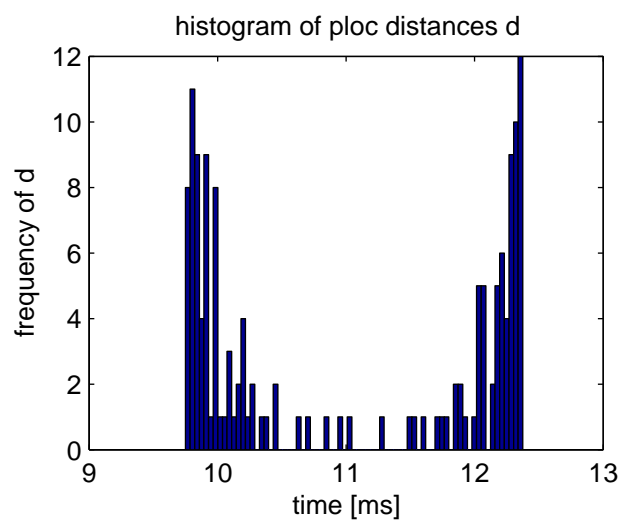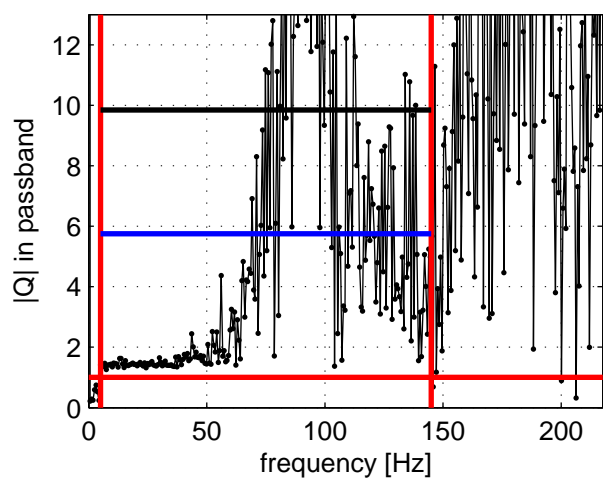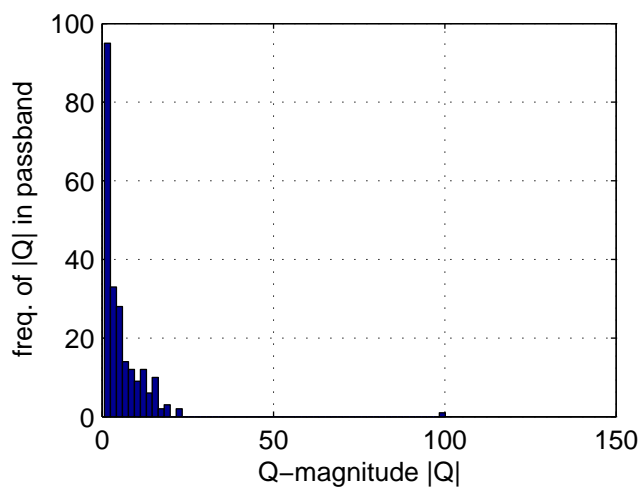

Supplement: Additional file 74 — q-sequence: 90persec_fig4_6 [file 1471-2202-7-18-S74.pdf]
